# Genome-wide analysis of the AP2/ERF family in *Musa* species reveals divergence and neofunctionalisation during evolution

**DOI:** 10.1038/srep18878

**Published:** 2016-01-06

**Authors:** Deepika Lakhwani, Ashutosh Pandey, Yogeshwar Vikram Dhar, Sumit Kumar Bag, Prabodh Kumar Trivedi, Mehar Hasan Asif

**Affiliations:** 1 CSIR-National Botanical Research Institute, Council of Scientific and Industrial Research (CSIR-NBRI), Rana Pratap Marg, Lucknow-226001, INDIA; 2 Academy of Scientific and Innovative Research (AcSIR), Anusandhan Bhawan, 2 Rafi Marg, New Delhi-110 001, India

## Abstract

AP2/ERF domain containing transcription factor super family is one of the important regulators in the plant kingdom. The involvement of AP2/ERF family members has been elucidated in various processes associated with plant growth, development as well as in response to hormones, biotic and abiotic stresses. In this study, we carried out genome-wide analysis to identify members of AP2/ERF family in *Musa acuminata* (A genome) and *Musa balbisiana* (B genome) and changes leading to neofunctionalisation of genes. Analysis identified 265 and 318 AP2/ERF encoding genes in *M. acuminata* and *M. balbisiana* respectively which were further classified into ERF, DREB, AP2, RAV and Soloist groups. Comparative analysis indicated that AP2/ERF family has undergone duplication, loss and divergence during evolution and speciation of the *Musa* A and B genomes. We identified nine genes which are up-regulated during fruit ripening and might be components of the regulatory machinery operating during ethylene-dependent ripening in banana. Tissue-specific expression analysis of the genes suggests that different regulatory mechanisms might be involved in peel and pulp ripening process through recruiting specific ERFs in these tissues. Analysis also suggests that MaRAV-6 and MaERF026 have structurally diverged from their *M. balbisiana* counterparts and have attained new functions during ripening.

Plants are sessile organisms and experience various stresses throughout their development. To combat these stresses, plants have evolved various mechanisms and processes for their survival and adaptation^1^,^2^. Since the domestication of crops, plants with better stress responsive traits and higher yield, sometimes compromising one character over the other, have been selected and multiplied^2^,^3^,^4^. To manifest various stress responsive processes, plant have evolved many large gene families -which sense and transduce the signals leading to modulated gene expression to combat stress at the molecular and cellular levels^1^,^4^,^5^,^6^,^7^,^8^,^9^. The AP2/ERF (APETELLA2/Ethylene Responsive Element Binding Factor) super family is one such gene family which is known to play a major role in the manifestation of the stress response in plants^4^,^5^,^6^,^7^,^10^,^11^,^12^,^13^,^14^.

The AP2/ERF domain containing gene family is one of the largest transcription factor families in plants. Till recently, this gene family was believed to be part of the plant kingdom only however; recent reports suggest presence of the AP2/ERF family members in various protists and ciliates^6^,^15^. The AP2/ERF transcription factor family is further subdivided into three (ERF, AP2 and RAV) families^5^,^6^,^12^,^14^,^16^. Most of the genes with a single AP2 domain and lesser number of introns are classified into ERF family. Members with tandem duplicated AP2 domains as well as single AP2 domain that differ from the ERF AP2 domain are classified as AP2 genes. Genes with AP2 domain and associated with B3 DNA binding domain have been classified as RAV (Related to ABI3/VP1) genes^17^. In addition, there is a small group of genes with a highly diverged single AP2 domain and gene structure known as the Soloists. Different subfamilies of this large gene family participate in modulation of expression of specialized genes^11^.

The ERF gene family has been studied in detail in many plants including those bearing climacteric and non-climacteric fruits^15^,^16^,^18^,^19^,^20^,^21^,^22^,^23^,^24^,^25^. This gene family is known to be involved in various abiotic and biotic stress responses^2^,^4^,^6^,^10^,^11^,^12^,^13^. Even though ethylene does not directly regulate all the members of this family, the name ERF (Ethylene Response Factor) has been carried forward due to their involvement in ethylene-regulated responses. Studies suggest that expression of these genes is modulated even in the absence of ethylene during various stress conditions and growth. Based on their gene structure and phylogeny, members of this gene family have been further subdivided into ten groups^13^,^16^.

The AP2 domain of 60 amino acid residues forms three anti-parallel beta sheets followed by a parallel alpha helix^6^,^15^. The first AP2/ERF domain was identified in APETALA2 of Arabidopsis^26^ and EREBP1 of tobacco^27^. The AP2/ERF domain includes two regions: the YRG region (YRG element) of about 20-amino acids, rich in basic and hydrophilic residues, in the N-terminal region. Various studies have validated participation of this region in binding through a direct contact with the DNA due to its basic character^5^. The second region is the RAYD sequence of about 40 amino acids. This region contains 18 amino acids capable of forming an amphipathic α-helix in the C-terminal sequence and is thought to have an important role for the structure and function of the domain^5^,^26^. The RAYD element was proposed to mediate protein-protein interactions through α-helix or to have an alternative role in DNA binding through interactions of the hydrophobic face of the α-helix with the major groove of DNA. AP2 domain containing transcription factors have also been classified as activators or repressors depending on whether they positively or negatively regulate expression of the target genes, respectively^6^,^28^. The activation domain identified in these transcription factors is not sequence-specific but generally contains acidic amino acid residues. Recently, the EDLL motif present in AtERF98/TDR1 transcription factor was shown to be a strong activation domain^29^. More than one repressor domain associated motifs including EAR motif, the TLLLFR motif, and the B3 repression domain (BRD) have been identified in AP2 domain containing transcription factors^6^.

AP2/ERF transcription factors regulate different development processes as well as stress response in plants. Therefore, this gene family has been characterized in detail in various plants^16^,^18^,^20^,^25^,^30^. Since various studies suggest involvement of this gene family in fruit ripening, it will be interesting to study the divergence of this gene family using ripening and non-ripening fruit varieties. Banana and plantains are the earliest cultivated crop plants. Bananas are generally consumed as fruits and plantains as vegetable and both are a rich source of nutrition for a majority of the world population^28^,^31^. The fruits of *M. acuminata* ripen and are climacteric i.e. require ethylene for ripening whereas the fruits of *M. balbisiana* do not ripen^28^,^32^,^33^,^34^. The plantains are sturdy and combat biotic and abiotic stresses better as compared to banana and hence are generally used for breeding purposes^35^. This suggest that AP2/ERF gene family might have undergone evolution during the divergence of banana and plantains to acquire specific traits like fruit ripening and stress adaptation as well as tolerance. In this study, we analysed the divergence of the AP2/ERF gene family and the emergence of new or neofunctionalisation of AP2/ERF genes related to ripening.

## Results

### Identification and phylogenetic analysis of AP2/ERF members in *Musa* species

A total of 285 and 325 putative AP2/ERF genes were identified from *M. acuminata* and *M. balbisiana* respectively by HMM blast. After removal of redundant genes and truncated sequences, this number reduced to 265 and 318 in *M. acuminata* and *M. balbisiana* respectively (Additional File 1). Further analysis using Interproscan software confirmed presence of the AP2 domain in the identified genes. To identify sequence similarities among AP2/ERF members, identified proteins of *M. acuminata* and *M. balbisiana* were aligned separately using Clustalx software. Alignment analysis suggests that ERF proteins of both species consist of 2 conserved regions, YRG and RAYD, in AP2 domain. Though overall sequence similarity between two species was 80 to 90%, sequences were of different lengths, isoelectric points (pI) and molecular weights in both species. The lengths of identified protein varied from 108 to 1029 amino acids in *M. acuminata* and 101 to 1040 amino acids in *M. balbisiana*. The pI of identified proteins ranged from 4.5 to 12.3 and 4.2 to 11.2 in *M. acuminata* and *M. balbisiana* respectively. Analysis also revealed that molecular weight of the polypeptides ranged from 12 to 114.8 kDa in *M. acuminata* and 11.2 to 118 kDa in *M. balbisiana* (Additional File 1).

**Figure 1:**
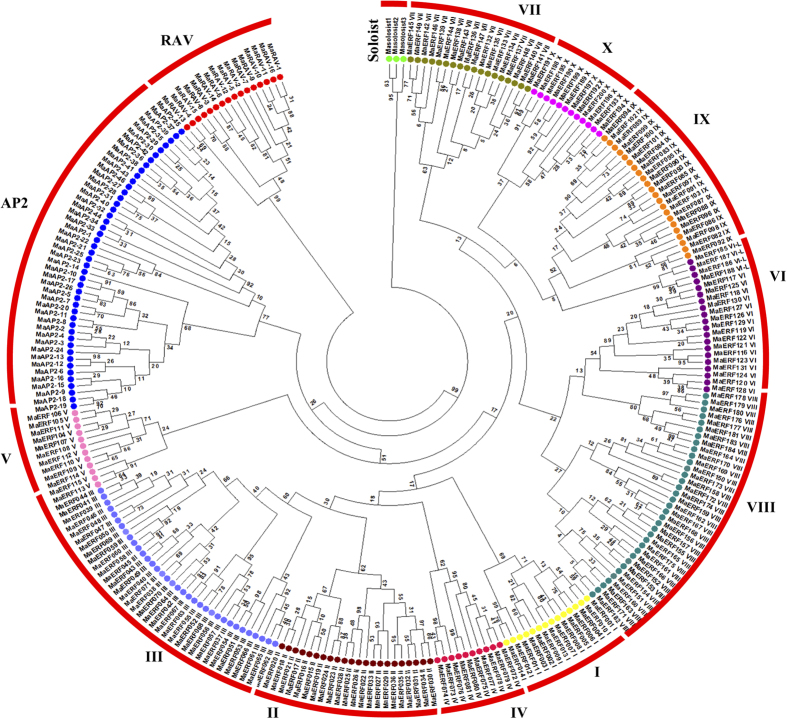
An unrooted phylogenetic tree of banana AP2/ERF proteins from *Musa acuminata*. Full-length domain of AP2/ERF proteins was identified and phylogenetic tree was constructed using Maximum likelihood method with 1000 bootstraps. Different groups of ERF family, AP2 and RAV family are divided with broken line.

A total of 265 and 318 sequences of AP2/ERF super family of *M. acuminata* and *M. balbisiana* respectively were used for phylogenetic analysis separately. The phylogenetic trees were constructed on the basis of ML method with a bootstrap value of 1000 using MEGA5.2. The phylogenetic analysis revealed that the AP2/ERF genes are further divided into four subfamilies, AP2, ERF, RAV and Soloist. In *M. acuminata*, the ERF subfamily with 205 members is further divided into ten subgroups. Of these subgroups, I to IV belong to DREB class whereas V to X belong to ERF classes based on the presence of the conserved amino acids at the 14^th^ and 19^th^ positions in the protein sequences. The ERF class contained 119 members having alanine and aspartic acid residues at 14^th^ and 19^th^ positions. The remaining 81 members contained valine and glutamine amino acid residues at respective positions and were classified as DREB (Supplementary Fig. S1). A total of 67 members contained double domain and were classified as members of AP2 and RAV families^7^. Our sequence alignment analysis suggests that 26 members contain 10 extra amino acid residues in first AP2 domain and are classified as ANT (AINTEGUMENTA) subfamily.Phylogenetic analysis revealed that the members with ANT-AP2 double domain and AP2-AP2 double domain are grouped separately. Similarly, members having AP2 and B3 domain grouped in separate clade (Fig.1). In our analysis, 20 genes with two consecutive AP2 domains were considered as members of the AP2 subfamily.

In *M. balbisiana,* out of 318 members, 243 and 71 genes contain single (ERF family) and double domains respectively. Of these, 22, 23 and 26 belong to the RAV, AP2 and ANT subfamilies, respectively. In addition, out of 243 single domain ERFs, 99 contain valine and aspartic at the 14^th^ and 19^th^ position and belong to the DREB subfamily. Remaining 144 proteins contain alanine at the 14^th^ position and belong to the ERF subfamily (Supplementary Fig. S2). Three and four genes were classified as soloist in *M. acuminata* and *M. balbisiana* respectively. On the basis of the group wise distribution, it can be observed that there is a remarkable increase in the number of genes in group IV and group X in case of *M. balbisiana* with an overall increase in the members of AP2, RAV and Soloist genes (Additional file 1). In comparison to other species, *Musa* has higher number of ERF genes and this increase is observed in all the groups. Similar to *M. acuminata*, there was no Xb like genes are present in *M. balbisiana* (Table 1).

### ERF groups in *Musa* species

On the basis of phylogenetic analysis, ERF genes in both the species could be clearly divided into 10 known groups (Fig. 1 and Supplementary Fig. S2). Interestingly, no member was found in Xb-L group in both the species. Detailed information related to members present in each group in both the genomes is provided in Table 2. Our analysis of the group I suggests that 4 more genes for this group are present in *M. balbisiana* as compared to *M. acuminata*. All other genes present in this group in *M. balbisiana* have orthologs in *M. acuminata*. Out of 4 additional genes, MbERF013 and MbERF014 have diverged from MbERF006, an ortholog of MaERF008. In addition, MbERF012 does not have any ortholog in *M. acuminata* indicating a gene loss in *M. acuminata.* (Supplementary Fig. S3). The other extra gene that is present in *M. balbisiana* in this group is MbERF011, a possible duplicon of MbERF009.

In group II, *M. balbisiana* contains five additional genes as compared to *M. acuminata*. Out of these extra genes, MbERF041 seems to have lost an ortholog as the other closest relatives of these genes have an ortholog in *M. acuminata*. Analysis also suggests that MbERF043 and 044 are duplications of MbERF031. Similarly, MbERF039 and 045 have either lost a counterpart in *M. acuminata* or have duplicated and diverged from MbERF034 in *M. balbisiana* (Supplementary Fig. S3). In group III, a large number of genes have either duplicated or diverged in *M. balbisiana* or their counterparts are lost from *M. acuminata* genome. At least 11 genes show divergence from their respective orthologs or homologs in this group. MbERF089 and MaERF064 have either diverged significantly or have lost an orthologs from their respective genomes. Interestingly, MbERF086, 085, 084, 080 and 083 have emerged new in the *M. balbisiana* genome. Analysis also suggests that MbERF071 and 077 are duplicons of MaERF067 as their ortholog. Group IV is the most conserved and there has been no change in *the* ERF genes between the two genomes (Supplementary Fig. S3).

In group V, only two extra genes are present, one each in *M. acuminata* and *M. balbisina*. MaERF108 has duplicated and diverged from MaERF107 and MbERF111 has duplicated and diverged from MbERF104 respectively (Supplementary Fig. S4). In group VI, there are two instances where a set of three genes have duplicated and diverged in *M. balbisiana*. Analysis suggests that MbERF128, 129 and 130 have duplicated and diverged from MbERF115. In addition, MbERF137, 138 and 139 have duplicated and diverged into VI-L group. Two new genes, MbERF127 and MbERF136, have duplicated and diverged or lost their *M. acuminata* counterpart (Supplementary Fig. S4). In group VII, two MbERF genes, ERF157 and ERF 149, have duplicated and diverged from their ancestral gene in this group. MbERF158 and MaERF145 seem to have evolved from the same ERF but diverged during evolution. A new duplication in *M. acuminata* has led to the emergence of MaERF141 (Supplementary Fig. S4). Group VIII has three evidences of genes that have duplicated and diverged in the *M. balbisiana* genome (MbERF 167, 192 and 191). MbERF195 and MaERF167 have diverged much during evolution. MbERF190 seems to be newly evolved as duplication of MbERF189. A new ERF in *M. acuminata* (MaERF161) has emerged, in this group, which is quite divergent from the other ERFs present in the same group. This could also be a case of lateral gene transfer in *Musa* sp. (Supplementary Fig. S4). There are two cases of gene divergence between *M. balbisiana* and *M. acuminata* genes in group IX. These include MbERF206 and MaERF084 as well as MbERF205 and MaERF089. MbERF221 has lost a counterpart in *M. acuminata*. Interestingly, our analysis suggests that there are seven cases in which *M. balbisiana* genes have lost their ERF counterparts in *M. acuminata*. These *genes* include ERF238, ERF237, ERF240, ERF239, ERF235, ERF243, and ERF236. In addition, MbERF241 and 242 were found as duplicated and diverged genes in the *M. balbisiana* genome (Supplementary Fig. S5).

### RAV and AP2

In RAV group, there is a presence of five extra genes in *M. balbisiana*. In addition, analysis suggests a loss of one gene from *M. acuminata*. Genes such as MbRAV15, 16, 11, 10, and 13 are duplicated and diverged from MbRAV-19 which have lost their *M. acuminata* counterpart (Supplementary Fig. S5). In AP2 group, MbAP2-14 and 16 are either newly emerged or have lost their *M. acuminata* counterparts. MbAP2-26 forms a separate branch from its *neighboring* genes suggesting its putative incorporation from lateral gene transfer (Supplementary Fig. S5).

### Diversity in gene structure shown in *Musa* species

Genomic structure analysis clearly suggests remarkable differences between ERF genes in *M. acuminata* and *M. balbisiana* (Additional file 2). In general, the ERF genes are classified as intron less or with few introns. Surprisingly in *Musa* species, number of introns is much higher as compared to other plants. In case of *M. balbisiana,* out of 318 genes, 119 contain introns. This number is much higher in *M. acuminata* in which out of 265 genes, 176 contain introns. This increased frequency of the intron occurrence could be due to the presence of higher number of transposable elements in the *M. acuminata* genome^36^. In Arabidopsis, out of 122 ERF genes, only 20 harbor introns. Similarly, in Rice out of 139 genes only 41 contain introns^15^ (Additional file 2). It is known that introns in ERF genes harbor transposable elements (TE) which could have played important roles during events leading to whole genome duplications and rearrangements. These TE and duplication events may have contributed to the increased number of introns as well as increased number of genes in the *Musa* species.

### Divergence of genes between *Musa acuminata* and *Musa balbisiana*

**Figure 2:**
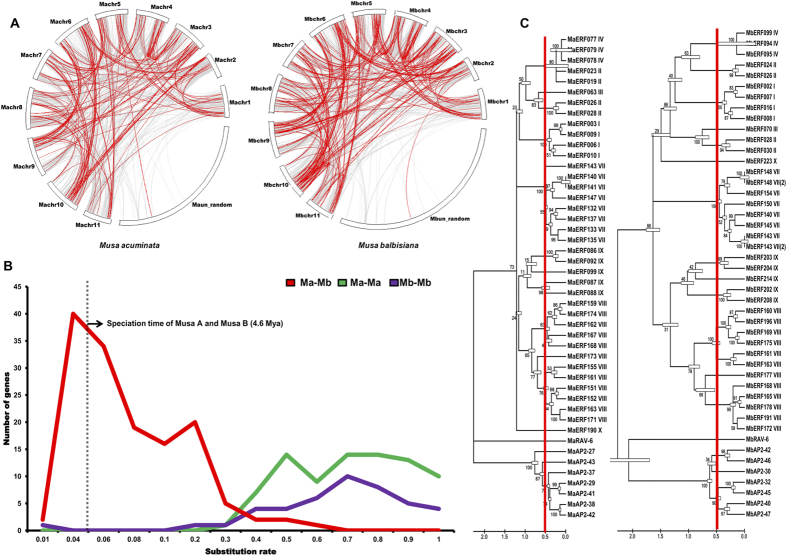
Synteny conservation of duplicated AP2/ERF genes and speciation of ERF gene in *Musa* genomes. (**A**) Dark red lines indicate duplicate ERF genes and Grey lines indicate collinear blocks within the A and B genome. (**B**) Red line indicates the substitution rate of ortholog ERF genes between the A and B genome. Vertical grey bar indicates speciation tine between the genome (4.6 million years ago). Green and Purple lines indicate the duplication event of ERF genes in A and B genome respectively. (**C**) Phylogenetic tree of diverged ERF genes involved in ripening process. Branch length in each phylogenetic tree is proportional to the synonymous substitution rate. The vertical red line bar in each phylogenetic tree indicates speciation time. Right and left trees show duplication event in A and B genomes, respectively.

To study divergence of genes, synteny analysis between the ERF gene family in *M. acuminata* and *M. balbisiana* was carried out (Fig. 2). Similar to Jourda *et al.*^37^, our study suggests that WGD is majorly responsible for the expansion of the ERF gene family in *M. acuminata* and *M. balbisiana*(Fig. 2a). The other changes have been brought about by genome rearrangements and losses during the speciation of *M. acuminata* and *M. balbisiana*. Though there is high level of synteny in the ERF genes between the two species, clear losses and duplications can also be observed. Only 232 ERF genes have homologs in both the species, the other genes *have* lost a counterpart in one of the species or some have emerged as a result of duplication events (Additional file 3).

Even though the genome of *M. balbisiana* (B genome) is smaller than the *M. acuminata* (A genome), it has higher number of ERF genes. To find out the reason for this, speciation time of orthologs AP2/ERF genes between A and B genome was estimated. The speciation time of A and B genome and Ks value (substitution rate) for the duplicated genes were reported as 4.6 Mya (Ks = 0.04) and Ks = 0.55 (61 Mya) respectively^28^. We analyzed peak value of Ks frequency of *orthologs* ERF genes at a substitution value of 0.02 (2.2 million years ago) (Fig. 2b). It indicated that AP2/ERF genes were diverged after the speciation of A and B genome. Further, we calculated the substitution rate of duplicated genes within the species and analyzed that the distribution of Ks around the 0.55. This suggests that large scale genome duplications have occurred in the *Musa* genomes much before the speciation of the A and B genomes of *Musa* with substitution rate of 0.04^28^. We speculated that around this time, events leading to α and β duplication may have occurred (Fig. 2c). Our analysis suggests the numbers of genes that have duplicated in *M. acuminata* are significantly much higher as compared to *M. balbisiana* (Fig. 2a). So, it could be possible that these genes, within the A genome, may have duplicated and diverged more as compared to their counterparts in the B genome.

**Figure 3:**
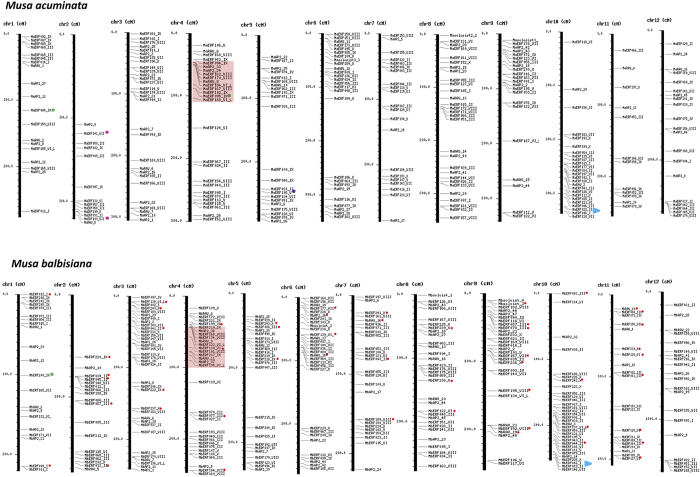
Chromosome localization of AP2/ERF genes on *Musa* genomes. Physical maps show the position of ERF genes on chromosomes of A and B genome separately. Horizontal grey line is representing the location of each ERF gene. The chromosome number is indicated at the top of each chromosome. Upper and lower panels show the distribution of ERF genes in A and B genomes respectively. Red dots in B genome indicated the ERF counterparts which are not present in A genome. Green dot shows gene loss in B genome. Red box and skyblue triangle shows the rearrangement region of AP2/ERF genes in both the genomes.

In addition, analysis also suggests that gene losses are more in *M. acuminata* as compared from *M. balbisiana*. This is also in agreement with the earlier report suggesting a large number of genes duplicated in recent WGD and have been lost in *Musa* genome^36^. So, it could be possible that the higher number of genes, in the B genome, is mostly due to the loss of their homologs in the A genome (Fig. 2a). To confirm the loss of genes in A genome, group wise phylogenetic analysis as well as chromosome localization of ERF proteins of both species was carried out (Fig. 3). Analysis, in both the cases, confirms that none of the genes contains duplicons elsewhere in the B genome. The only evident case of duplicons was the MbERF011/MbERF009 and MbERF070/MbERF077. Loss of these genes could be due to large scale genome rearrangements in the A and B genomes. Analysis also suggests loss of a homolog (MaERF108) from the B genome as compared to A genome in only one case. There are two cases in which the genes in A and B genome are not present in the same position like MaERF068 and MbERF082. MaERF068 is present on chromosome 8 of A genome whereas MbERF082 is present on chromosome 11 of B genome. Similarly, MaERF095 is present on chromosome 4 and its homolog MbERF200 is present on chromosome 1. Interestingly, it seems that during transfer of MaERF095 from chromosome 1 to chromosome 4, a copy of MaERF084 was left on chromosome 1.

### Presence of conserved motif analysis other than AP2 domain

Apart from the conserved AP2 domain, the ERFs have conserved domains specific to various groups. These motifs participate in transcriptional activities and protein-protein interactions. In our analysis, a total of 27 and 35 motifs are identified in *M. acuminata* and *M. balbisiana* proteins encoded by this gene family respectively (Supplementary Tables S1 and S2). Though group-specific motifs are conserved in the subfamilies of ERF, some differences have been reported in motifs present in various plants in different groups of ERF family 15,16. The conserved motifs in the ERFs, of both the species, also show significant differences which might be responsible for the functional differences in the ERFs from both the species. Some of the identified known motifs -and their structural differences present in both the species are summarized in Supplementary Tables S1 and S2. Most of the proteins in each groups shared similar motifs. For instance, KYPS and LNFP motifs were identified in the C-terminal of proteins belong to group-I in both species respectively^15^. The protein sequences of Group III, Group IV and Group V shared different motifs while Group VII have similar conserved motif pattern in both the species.

WDE motif present in group I is known to be involved in transcription regulation and act as histone acetyl transferases^16^. In addition, the KYPS motif is involved in methylation and plays an important role in regulation of genes in developmental processes 15,16. The LNFP is group II specific and known to presents in disease resistance genes^15^,^39^. These set of ERF genes may be involved in providing resistance to pest attack. The LPR[P/A], D[I/V]QAA, [L/R]AAA motifs are essential signatures in Arabidopsis ERFs which function as CBL-interacting serine/threonine protein kinases^15^,^16^. Involvement of these genes in various signaling pathways in banana cannot be ruled out. The motif SP(T/V)SVL present in group VI is a MAPK which is a potential phosphorylating motif 15,16. The motifs (MCGGA and DFEADF) present in group VII are highly conserved and are known to participate in ethylene transcriptional activation signaling cascade^15^,^16^. The other motifs like RG[k/s/r]KAKVNF, TNF, LNFP and LWSFD are also present in other ERFs and ERF-like proteins and may be participating in various molecular networks and processes involved in plant growth and stress response. The EAR motif, present in group VIII genes, is known to be present in the repressor type of ERF proteins^15^,^40^. Apart from the above discussed common motifs present in both *M. acuminata* and *M. balbisiana*, there are several others motifs that are present only in *M. balbisiana*. These motifs may be involved in DNA interactions or in the structural confirmation of proteins (Supplementary Tables S1 and S2).

### Differential expression of ERFs during fruit ripening

**Figure 4:**
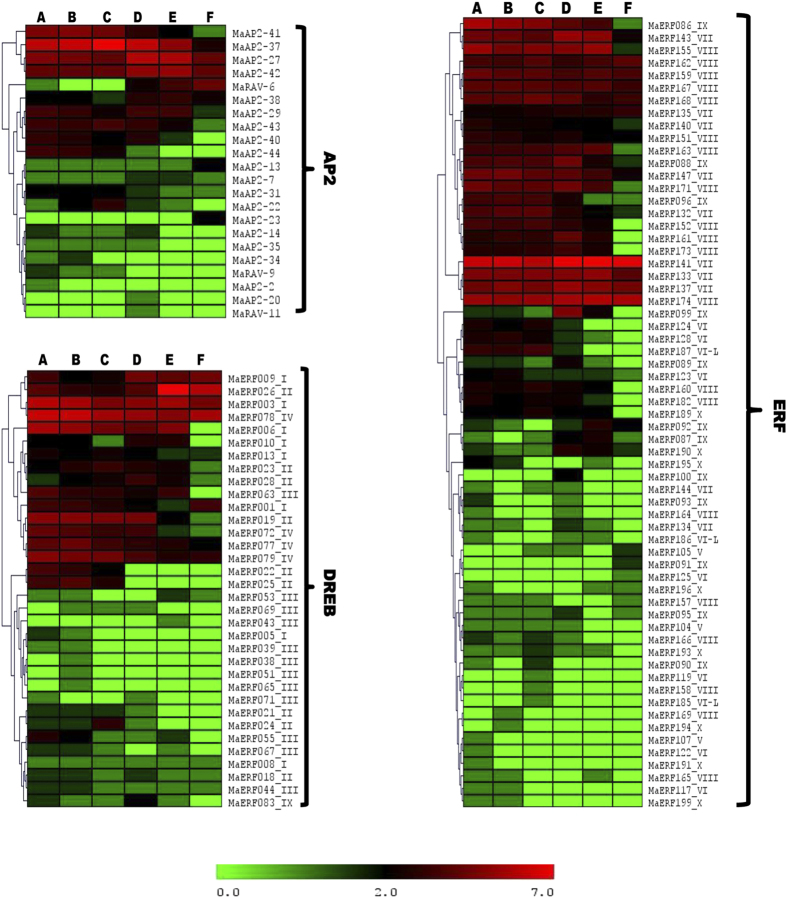
Expression of AP2/ERF genes in banana fruits in response to ethylene. Log_2_ transformed count value is used for heat map construction. In top of heat maps A, B and C indicate expression of genes in response to without ethylene treatment and D, E and F indicate expression of genes in response to ethylene treatment across three time point (40 days, 60 days and 90 days).

To identify ethylene responsive AP2/ERF genes, the expression of ERF genes was analyzed using RNA seq libraries of banana fruit (cv. ‘Cavendish’, AAA triploid)^36^,^38^. From the pool of 265 members of ERF gene family present in *M. acuminata*, 122 genes showed the presence in the libraries^36^,^38^ of banana fruit, indicating their possible involvement in the fruit development and ripening (Fig. 4). In our earlier study using genome-wide transcriptome analysis of ripe and unripe fruit of banana (Dwarf Cavendish, Genome AAA, var. Robusta, Harichhal, germplasm code TRY0081 at National Research Centre for Banana, India), 81 ERFs were identified^38^. Of these, 71 ERF genes were present in both transcriptome data sets (Supplementary Fig. S6). It seems that that these genes might be involved in fruit development and ripening process across the varietal differences of *M. acuminata*. From these 71 ERF genes, there are nearly 40 ERF genes which were significantly differentially expressed between ripe and unripe fruit of banana on the basis of normalized expression value in RNA-seq data. It suggests that these identified ERF genes are fruit-specific and ripening-related. Further, we identified homologs of ERF genes in *M. balbisiana* using reciprocal blast. Interestingly, though the average similarity between the homologs of both genomes is up to 90%, some of the genes involved in ripening in the Cavendish variety have similarity as low as 80%. This suggests that changes in sequences may have resulted in their neofunctionalisation for their involvement in ripening and other processes. Of the 40 ERF genes in *M. acuminata,* which are putatively involved in ripening, only 27 have similar homologs in *M. balbisiana*. The other thirteen genes have highly diverged homologs as per analysis carried out through reciprocal blast.

To further analyze these 40 ERFs from *M. acuminata*, structural comparisons between homologs in *M. balbisiana* was carried out. Out of these 40 genes, few have diverged significantly from their homologs and form a significantly different structure. This structural change may be more receptive to the MAPK pathway and play an important role in the ethylene perception and signaling cascade. Of these, MaRAV-6 showed significant increased expression during ripening in *M. acuminata* (varieties Harichal and DH Pahang) in our analysis. The structural analysis of MaRAV-6 showed that though polypeptide encoded is similar, it forms different structure and does not superimpose well with their homologs in *M. balbisiana*. This raises a possibility for the different functions of these genes during ripening. As *M. balbisiana* is a non-ripening variety^31^,^35^, MbRAV-6 gene may not be involved in any of the ethylene or ripening regulated pathway.

### Expression analysis during fungal attack

The expression of the ERF genes from *M. acuminata* was studied in the fungal disease data sets available in public domain^41^. In this study, the corms of the resistant variety NK and susceptible variety BK were treated with fungus *Fusarium oxysporum* for 48 and 96 h. All the genes, except 41 ERF genes, showed their presence in these datasets. This suggests that these 41 ERFs might be expressed in other tissues and/or in response to other stresses. Analysis also suggested that a large number of ERF genes are differentially expressed in response to pathogen attack (Supplementary Fig. S7) in these two varieties. The differential expression of these ERFs might be due to specific response to the pathogen stress in these two varieties.

### Tissue-specific expression pattern of ERF genes

It is reported that ethylene plays an important role in the regulation of banana fruit development and ripening process. This process is known to modulate the expression of several genes including ERF transcription factors. These ERFs are also involved in many development and cellular processes such as response to abiotic and biotic stress, regulation of metabolism, flower and fruit development, growth and many more in various plants^6^,^11^,^13^. In our study, the expression of a set of ERFs in different tissues and ripening process was studied by qRT-PCR. For this, nine ERFs were selected because of their presence in RNA seq datasets of D’hont^36^ and Asif *et al*.^38^. Apart from these nine ERFs, five more ERF genes were selected from D’hont^36^ data sets due to their exclusive presence in this data set. These ERFs were selected to generate information about varietal specific ERF expression between the two varieties. Though fold change in the expression of the selected genes between D’hont^36^ and qRT-PCR, carried out in this study, was not similar, trend in up- and down-regulation of genes was same in both the experiments.

**Figure 5:**
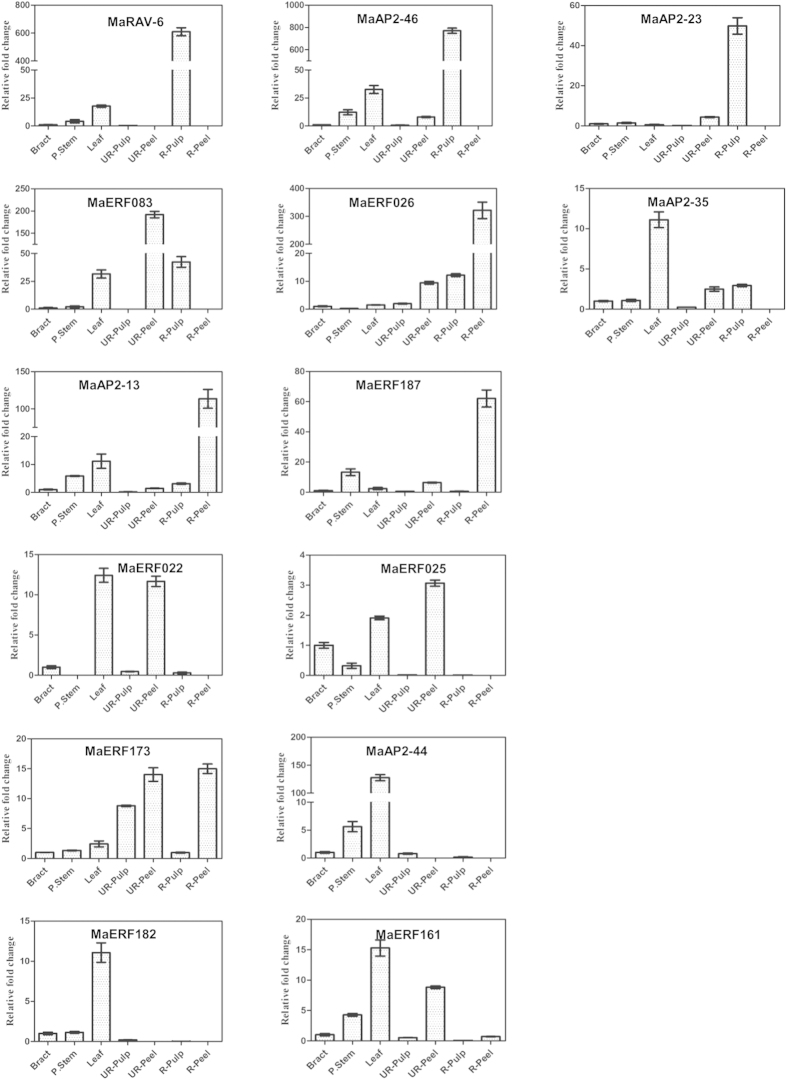
Tissue specific expression pattern of AP2/ERF genes of *Musa acuminata*. Total RNA isolated from different tissues (Bract, Psudostem and Leaf) as well as peel and pulp tissues of ripening and non-ripening banana fruit. Expression level was calculated using qRT-PCR.

The expression analysis for fourteen ERFs was carried out in bract, stem and leaf tissue as well as peel and pulp tissues of ripening and non-ripening banana fruit (Fig. 5). It was observed that MaRAV-6, MaAP2-46, MaAP2-23 and MaERF083 genes are highly expressed whereas MaAP2-35 is minimally expressed in ripe pulp tissue. The expression of MaAP2-23 was observed only in pulp tissue and not in any other tissue. On the other hand, MaERF026, MaAP2-13, MaERF087 and MaERF173 were highly expressed in ripe peel tissue as compared to other tissues. It was reported earlier that different regulatory mechanism is involved in peel and pulp tissue during ripening process^42^. Therefore, it could be possible that different ERFs might be involved for regulating ripening process in peel and pulp tissues.

All the MaERFs selected for the analysis showed expression in leaf and stem tissue, except MaAP2-23 in leaf and MaERF22 as well as MaERF26 in stem. MaRAV-6, MaAP2-35, MaERF083 and MaAP2-46 genes were expressed in leaf tissue in addition to ripen pulp tissue. It indicates that genes might have a possible role in ripening and leaf development processes. In bract tissue, MaRAV-6, MaAP2-46, MaAP2-23, MaERF083 and MaERF187 genes were not significantly expressed. Result indicated that the MaERF025, MaAP2-44, MaERF182 and MaERF161 genes express in different tissue. Interestingly, ERFs which were present specifically in D’hont^36^ dataset were highly down-regulated in ripening process. It indicates that these genes are not involved in ripening process and might be involved in other development processes.

### Expression profiles of ERFs in dessert and cooking varieties

**Figure 6:**
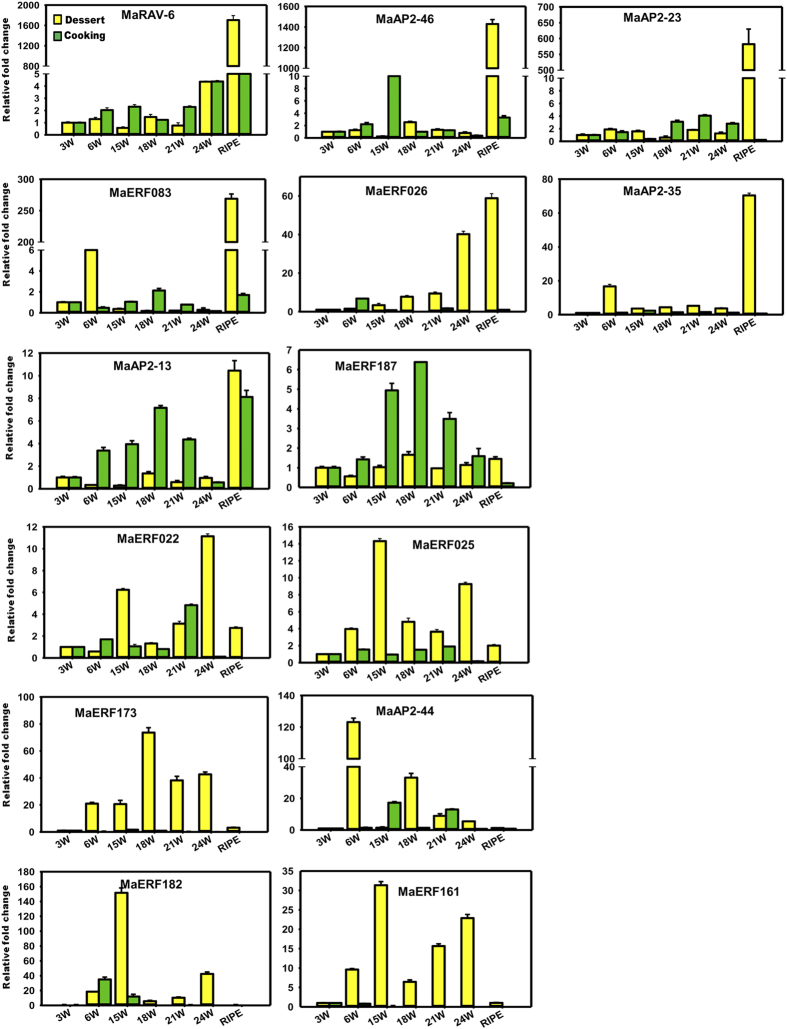
Modulation of gene expression in different fruit developmental stages and ripe fruit tissues. Expression level is expressed as relative fold change as compared to the tissue with the lowest expression level. Specific-primer set of individual genes was designed from intron spanning region. Yellow bar indicate the expression value in *M. acuminata* (dessert variety; AAA genome) and green bar indicate the expression value in *M. paradisiaca* (cooking variety; AAB genome).

Dessert (*M. acuminata)* and cooking (*M. paradisiaca)* varieties of banana were used to identify the possible role of ERFs in fruit development and ripening process. *M. paradisiaca* is the hybrid of *M. acuminata* and *M. balbisiana* belonging to AAB genome^43^. Expression analysis of 14 selected ERFs was carried out in different fruit developmental stages (3W, 6W, 15W, 18W, 21W and 24W) and ripening stage of pulp (Fig. 6). Most of genes showed contrasting expression pattern in two varieties. MaRAV-6, MaAP2-46, MaAP2-23, MaERF083, MaERF026, MaAP2-35 and MaAP2-13 genes were highly expressed in pulp of ripe fruit as compared to fruit development stages. Interestingly, MaAP2-35 was specifically expressed in dessert variety where as RAV-6 gene showed highly dissimilar expression in both varieties. The expression patternof MaAP2-46, MaAP2-23, MaAP2-13, MaERF187 and MaERF022 genes suggests that these genes were more involved in different fruit development stages as compare to ripening process in cooking variety. MaAP2-13 and MaERF187 were expressed during fruit development as well as in ripening process in the both varieties whereas MaER026 was highly expressed in ripening stage followed by 24W fruit development stage in dessert variety. Interestingly, expression of MaERF173, MaAP2-44, MaERF182 and MaERF161 genes was observed only in fruit development stages and not during ripening stages in both varieties. Expression analysis suggests that MaRAV-6, MaAP2-46, MaAP2-23, MaERF083, MaERF026 and MaAP2-35 ERFs are ripening specific genes.

### Structural analysis of ripening related ERF genes

**Figure 7:**
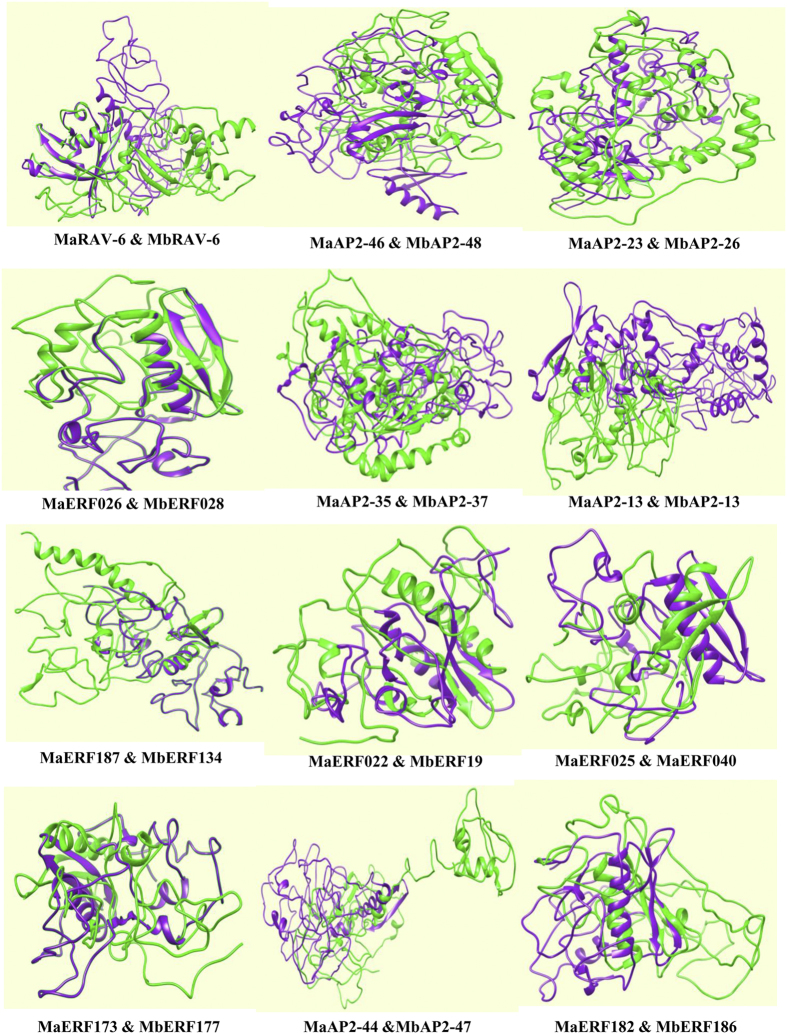
Structural predictions of AP2/ERF proteins. Proteins structures of fourteen ERFs of *M. acuminata* and their homologs in *M. balbisiana* were predicted using PHYRE2. Proteins in green and purple colors indicate the structure of ERF proteins from *M. acuminata* and *M. balbisiana* respectively.

Function of proteins depends directly on their structure. Proteins belonging to the same family may contain similar active domain but due to their conformational changes may show different functional behavior. The protein sequences of the 14 ERF genes from *M. acuminata* and their homologs in *M. balbisiana* were used for the comparative structural analysis. Protein sequences of these ERF genes were modeled using PHYRE2 web server^44^. The structure of each MaERF protein was superimposed on its homolog in *M.balbisiana* to study structural divergence between ERF genes of both species. Interestingly, in most of the cases, proteins did not superimpose on their homologs in *M. balbisiana* due to conformational changes of proteins in both the varieties(Fig. 7). These conformational differences in the structure of proteins may have led to the neofunctionalization of the homologs (Fig. 7).

### Expression, Structural and evolutionary analysis of MaRAV-6 and MaERF026

**Figure 8:**
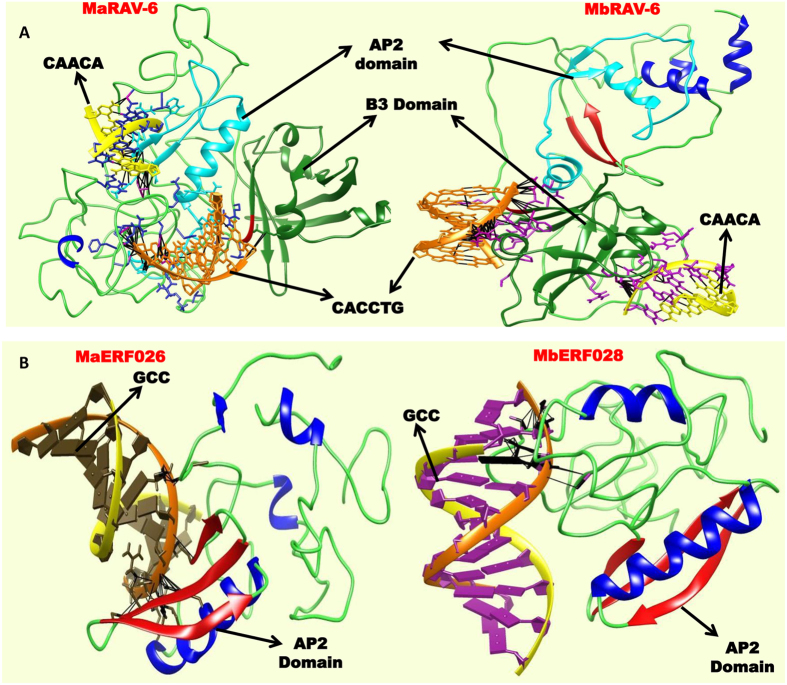
Docking structural analysis of MaRAV6 and MaERF026 with different motifs. (**A**) MaRAV-6 and MbRAV-6 show the interaction with CAACA and CACCTG DNA region. (**B**) MaERF026 and their homolog in *M. balbisiana* interaction with GCC DNA region.

Several of the ERFs identified showed ripening related expression in *M. acuminata.* Out of these, MaRAV-6 and MaERF026 genes showed functional and structural divergence from their homologs in *M. balbisiana*. Therefore, MaRAV-6 and MaERF026 genes were selected to explore the functional divergence and role in ethylene response. RAV and ERF transcription factors belong to different ERF families, recognize different *cis*-regulatory elements and regulate several genes involved in different biological processes. RAV genes contains N-terminal AP2 and C-terminal B3 domains which recognize “CAACA” and “CACCTG” motifs respectively. At the same time, ERF family recognizes the “GCC” DNA region in the target genes^17^,^45^. To explore the role of these two genes in ethylene response, protein-DNA docking of ERF protein with their respective DNA motif was carried out. Our analysis suggests that structural conformation of MaRAV-6 from is significantly different from MbRAV-6 as both are only partially superimposed to each other. It indicates that structure of MaRAV-6 might have diverged from their homologs in *M. balbisiana*. RAV family proteins function as transcriptional repressors due to existence of novel repression domain R/KLFGV in Arabidopsis^6^,^11^,^16^. The R/KLFGV motif was identified in RAV6 protein of both the species. It indicated that protein has repressor activity. Further, docking of RAV proteins with two motifs “CAACA” and “CACCTGG” revealed that AP2 domain interacted with CAACA whereas B3 domain showed less interaction with CACCTGG in *M. acuminata*. However, both CAACA and CACCTGG regions showed high interaction with B3 domain while AP2 domain did not show interaction with CAACA motif in *M. balbisiana* (Fig. 8a). These results suggests that the genes which have “CAACA” *cis*-elements in the promoter region of target genes might be differentially regulated by RAV6 in *M. acuminata*. Analysis also suggests that repressor activity of RAV6 is more in *M. balbisiana* than *M. acuminata*.

Expression analysis using different stages of fruit development and ripening process suggests that expression of RAV6 gene is significantly enhanced in pulp of dessert variety as compare to cooking variety during the ripening process (Figs 5 and 6). However, in cooking variety, MbRAV-6 is highly expressed as compare to dessert variety in fruit development stages. It indicates that MaRAV-6 might be involved in fruit ripening process in banana whereas MbRAV-6 may participate in fruit development process.

To identify the role of ERF026, we performed the structural analysis of MaERF026 and its ortholog MbERF028. Structural analysis suggests that though the AP2 domains of both proteins superimposed to each other, conformation of both proteins was significantly different. Docking of MaERF026 and MbERF028 with “GCC” motif in DNA region revealed that MaERF026 has significantly higher binding -affinity with GCC-rich region as compared to its ortholog gene in *M. balbisiana* (Fig. 8b). This ripening-related ERF from *M. acuminata* with higher GCC-rich domain binding capacity may be part of the ethylene signal transduction cascade involved in ripening process. As *M. balbisiana* ortholog does not show such binding pattern with GCC-rich domain, its function may be during banana fruit development process^35^. Analysis also suggests that these genes might have evolved and diverged from their orthologs and developed new functions. Tissue-specific expression analysis showed that MaERF026 gene highly expressed in ripe peel tissue than the pulp, stem, bract and leaf tissue. In addition, MaERF026 is highly expressed in ripen pulp tissue of dessert variety followed by 24 week fruit development stage. However, in cooking variety, it showed very less expression in ripe pulp tissue and highly expressed in 6 week fruit development stage. This result suggests that MaERF026 gene might be involved in banana fruit ripening (Fig. 8b).

### Proximal promoter analysis

It has been previously shown that the EIN3 binds the Ethylene-responsive element (ERE;CATAAGAGCCGCCACT) in the promoter of the ERF1 gene in *Arabidopsis* and leads to is activation for further ethylene responses^46^. In the present study, we analyzed presence of various *cis*-acting regulatory elements in the 1.5 kbp upstream region from the translation start site in all the ERFs identified in this study. Emphasis was given to analyze presence of 10 motifs including AuxRE, AuxRR-Core, BoxW1, Crepeat/DRE, DRE, ELI-box3, ERE, motif IIb, MRE and WUN-motif which are known to regulate expression of genes during hormonal signaling, dehydration, wounding and other biotic and abiotic stresses^47^. Interestingly, out of all the ERFs, promoter regions of 13 genes contained ERE motifs. Out of the ERE-containing ERF genes, 5 are present in the list of ripening related ERFs (Additional File 4). In addition, 9 of the 13 ERE motif containing genes also contain ARE (TGTCTC) motif which suggest their role in the possible cross-talk between auxin and ethylene hormones. The auxin response element was present in 86 of the total ERF genes in *M. acuminata*. Such auxin and ethylene cross talk has been demonstrated during ethylene induced banana fruit ripening^33^. Interestingly, fungal elicitor response element, the BoxW1 (TTGAC) was present in proximal promoter of 76 ERF genes. This element is known to participate in fungal response and might be playing important role during fungal infection in banana^47^. Surprisingly, other biotic and abiotic stress related motifs were present minimally in ERF proximal promoters. Out of these, DRE, WUN motif 1and C repeat/DRE were present in only one gene each out of all the ERFs.

## Discussion

The AP2/ERF gene family has been studied in detail in several plants including Arabidopsis, rice, populus, apple however, no detailed study has been carried out in *Musa* species^16^,^18^,^20^,^25^. This gene family plays an important role in various stages of plant development, stress tolerance and fruit ripening. In this study, the evolution and divergence of the AP2/ERF genes in *Musa* species was studied to identify specific and subtle changes in the genes resulting in sub and possible neofunctionalisation of the genes. A comprehensive analysis of AP2/ERF genes in *Musa* identified 265 and 318 genes in *M. acuminata* and *M. balbisiana* respectively (Additional File 1). The overall number of the AP2/ERF genes was high in the *Musa* species. and was the highest in monocots^16^,^18^,^20^,^22^,^24^,^25^. Though the largest AP2/ERF gene family reported till date was from *Brassica rapa*, our study suggests that *M. balbisiana* has more number of the members as compared to *B. rapa*. This increase in gene number in *Musa* species can be attributed to the three (α, β and γ) WGD duplication events that have occurred in *Musa* species after divergence from Zingiberales^36^,^37^. The γ duplication which occurred earlier corresponded to the σ duplication event in Poales and α, β duplication events occurred in a small span of time corresponded to the ρ duplicaton event in Poales. The AP2/ERF genes generally have very few intron containing genes. However the number of intron containing genes in *Musa* species was much higher as compared to other plants^16^. This increase in intron containing genes could be due to the higher number of transposable elements in the *Musa* genomes^36^,^37^ as compared to Arabidopsis and rice.

Phylogenetic analysis carried out in this study reveals that *Musa* species follow the same distribution pattern of ERF genes as in other plant species (Fig. 1 and Supplementary Fig. S1). Based on the previous classifications, the *Musa* AP2/ERF genes were classified as AP2, ERF, RAV and Soloist. The ERF subfamily was further divided into DREB (I-IV) and ERF (V-X) groups. The overall distribution of the genes was similar to other species, except a significant increase in the RAV gene members; the other plant species had less than 5% of the genes in RAV subgroup, whereas in *Musa* species it was >6% of the genes. Within the *Musa* species, *M. balbisiana* had higher percentage of DREB members and lesser percentage of ERF members as compared to *M. acuminata*. This can be correlated with the better stress tolerance level of *M. balbisiana* as compared to *M. acuminata*. The number of Soloist gene members was also higher in *Musa* species *as* compared to other monocots.

While having high degree of sequence conservation, significant variation in the gene structure of ERF genes was noticed between the two species. The AP2/ERF genes in *Musa* species have expanded due to WGD as in Arabidopsis in contrast to rice and Chinese plump^16^,^20^ in which largely tandem duplications have taken place. Most interestingly, the genes which were duplicated by the earlier, γ WGD, event were retained in *Musa* species as compared to the latest α, β duplications. It has also been reported that large scale genome rearrangements and losses have occurred in *Musa* species after the WGD events resulting in loss of genes and maintaining in gene balance. Analysis suggests that occurrence of these rearrangements and losses are more in *M. acuminata* as compared to *M. balbisiana* resulting in lesser ERF gene number in *M. acuminata*. The AP2/ERF genes of *M. acuminata* and *M. balbisiana* have diverged 2.2 Mya which is after the divergence of A and B genome and might have resulted in neofunctionalization or subfunctionalization of duplicated genes^28^,^37^.

Our analysis suggest that several ethylene responsive genes are retained after WGD to maintain a balance between genes involved in this pathway and also to develop fine control of ethylene signaling^37^,^49^. Most of the genes with high divergence might be involved in the ethylene responses in Cavendish variety and could be significantly diverged from their homologs in non-ripening variety. Some of the genes like MaERF141, MaERF099, MaERF137, MaERF135, MaERF010, MaERF161, MaERF018, MaAP2-34, MaAP2-31, MaERF167, MaERF063, MaERF159, MaERF171, MaERF132, MaERF006 have significantly diverged from their *M. balbisiana* orthologs after the A and B genome divergence. A few of these genes are also differentially expressed in ripening and non-ripening varieties indicating neofunctionalisation of these genes.

The transcriptome analysis suggests that some duplicated ERF genes present in A genome are differential expression during ripening. Such differentially expressed genes include up-regulated MaERF141 and down-regulated MaERF136 and 147 during ripening. There are several other duplicated genes which showed differential expression. Studies also suggest that several insertions and deletions have occurred during the period of divergence of two *Musa* species^36^. Several ERF genes in A genome may have lost their counterparts in B genome during these events. This was also confirmed through phylogenetic and chromosome localization analysis. These analyses revealed that several genes are lost in A genome leading to higher number in B genome (Fig. 3 and Supplementary Fig. S3).

Identification of various conserved motifs other than the AP2/ERF domain suggests that the distribution of motifs is specific to each class in the phylogenetic tree and as described in other plant species^5^,^6^,^16^,^27^,^40^. This indicates the structural similarities among the proteins within the groups similar to rice and Arabidopsis. Distribution of conserved motifs outside the AP2 domain in rice and *Musa* species shared mostly similar motifs regulating various biological processes. In addition, a short motif (NSGEPDPVRIKSKRS) which is specific to dicots is also absent in *Musa* species^16^. It indicated that monocots share lineage specific motif patterns.

Previous analysis of RNA-seq data suggested that 22 ERF members express in banana fruits (pulp tissue)^37^. Our analysis, in this study, suggest that 40 genes express during ripening process and might play important role in banana fruit ripening. In addition, functional redundancy between these members might exist as these also express during fungal infection and might be involved in other biological processes such as stress and defense response^5^,^7^,^11^,^13^,^27^. Through analysis of the expression of ERF genes of fruits with or without ethylene treatment, we concluded that the majority of fruit-specific ERF genes are down-regulated during the banana fruit ripening. It raises a possibility that ERFs might negatively control the ripening process in banana^37^,^42^. Previous studies suggested that ethylene biosynthesis is negatively controlled in pulp and positively controlled in peel tissue during ripening process^37^,^42^.

To validate the expression of genes during ripening and fruit development expression analysis of 14 selected genes was carried out in two species of *Musa*, the cooking variety (*Musa paradisiaca*; ABB genome) and the dessert variety (*Musa acuminata*; AAA genome). The expression analysis suggested differential expression pattern in different stages of fruit development and ripen pulp of banana fruit in both varieties. In this analysis, 9 genes were expressed either in pulp or peel of ripe banana fruit (Fig. 6). MaAP2-13, MaERF187 and MaERF173 genes are specifically expressed in the ripe peel tissue but not in pulp tissue. In addition to this, 4 genes (MaRAV-6, MaAP2-46, MaAP2-23 and MaERF083) were expressed specifically in the ripe pulp tissue. This suggests that differential regulatory mechanism might be involved during ripening process in peel and pulp of banana.

Our analysis also suggested that a set of genes(MaRAV-6, MaAP2-46, MaAP2-23, MaERF083) are highly expressed in ripe pulp tissue as compare to fruit development stages in dessert variety. In addition, same genes were lowly expressed in fruit development stages as well as ripe pulp tissue in cooking variety (Fig. 6). It indicates that these genes may be more involved in fruit ripening process. MaERF026 and MaAP2-35 genes exhibited significant higher expression in dessert variety as compared to cooking variety. This finding suggests that MaERF026 and MaAP2-35 might have distinct role which specific to in dessert variety. Moreover, MaERF026 gene expressed in both ripen peel and pulp tissue of banana (Fig. 5) suggesting functional redundancy of MaERF026 in peel and pulp tissue during the ripening process through sharing common mechanism to regulate the ripening process. The expression of MaAP2-13, MaERF173 and MaERF187 was higher in ripe peel tissue as compare to ripe pulp tissue of banana fruit (Fig. 5).It indicates that these genes followed different regulatory mechanism to regulate ripening and fruit development process.

RAV gene family is plant specific, act as transcriptional repressor and is known to be induced by ethylene^17^,^45^. Several other studies support that RAV members play an important role in plant development and growth process including leaf senescence. To gain insight into role of MaRAV-6 gene in the regulation of ripening process, expression profile was analyzed during the different fruit development stages of dessert variety as well as in ripen peel and pulp tissue of dessert and cooking variety. Our analysis confirms that MaRAV-6 has an important role in the regulation of ripening process. The expression of RAV gene is highly induced in dessert variety as compared to cooking variety during ripening process. This suggests that this gene might be play regulatory role through regulation of genes by the transcriptional activation or repression during the ripening process. In addition, our study also suggests that MaERF026 gene is highly up-regulated in ripen peel tissue in dessert variety during the ethylene response and might be involved in ethylene induced changes in peel tissue during ripening process.

In this study, identification and detailed analysis of a very diverse and important gene family, AP2/ERF, has been carried out. Analysis suggests that ERF gene family in *Musa* species has diverged during evolution leading to loss of many of the homologs due to genome rearrangements. In *M. acuminata*, some of the genes including MaRAV6 and MaERF026 have significantly diverged for ripening related functions. The ripening related changes in the *M. acuminata* species are basically due to subtle changes in sequences of the ERF genes which lead to changes in their structure and binding capacity.

## Methods

### Identification of members of Ap2/ERF super family

A total 1055 AP2/ERF proteins of 40 different plant species were downloaded from plant transcription database (http://planttfdb.cbi.pku.edu.cn/) and a HMMER profile of these AP2/ERF proteins was constructed for the identification of AP2/ERF members in *M. acuminata* and *M.balbisiana*. Protein and CDS sequences of *M. acuminata* and *M. balbisiana* were downloaded from banana genome hub (http://banana-genome.cirad.fr/)^50^ and Hmmer profile as database was run against protein sequences of both species using the HMMER v3.1b2 software (http://hmmer.janelia.org/)^48^. To confirm the AP2 domain in identified proteins, domain analysis was performed using Interproscan tool (http://www.ebi.ac.uk/Tools/pfa/iprscan5/). For the further analysis, genome of both species was downloaded from banana genome hub.

### Alignment and classification of AP2/ERF protein sequences

To identify the AP2 domain in the protein sequences of banana, multiple sequence alignment was carried out using ClustalX program (http://www.clustal.org/)^51^ and AP2 domain was highlighted in aligned protein sequences using the Box shade program (http://www.ch.embnet.org/software/BOX_form.html). The Phylogenetic approach was used for the classification of AP2/ERF members. AP2/ERF protein sequences of rice and banana species were aligned with each other using ClustalW program. These aligned sequences were used for the construction of phylogenetic tree using Maximum likelihood method^51^. The unrooted tree was constructed with 1000 bootstraps using MEGA software (http://www.megasoftware.net/)^52^.

### Gene structure and chromosome location analysis

AP2/ERF CDS sequences were mapped against banana genome using BLAST tool to identify the genomic location of each gene members on chromosomes. Then genomic sequences of identified AP2/ERF genes were retrieved using custom Perl script. Further gene structure display server was used to determine the exon-intron boundaries^53^. Physical mapping of identified genes on the banana chromosome was done using custom Perl script.

### Evolutionary and gene expansion analysis

To investigate the evolutionary mechanism in AP2\ERF family, we identified the orthologs genes of identified AP2\ERF genes between *M. acuminata* and *O. sativa* as well as *M. acuminata* and *M. balbisiana* using reciprocal blast with e-value 10^−5^ According to the reciprocal blast output, duplication events were identified using the McScanX software^54^. Ka/Ks analysis of orthologs and paralogs sequences was done using PAL2PAL and Codeml program^55^. Time (million years ago, Mya) of duplication and divergence was calculated using a synonymous mutation rate of substitutions per synonymous site per year as T = Ks/2 λ. (λ = 4.6*10^−9^)^28^.

### Expression analysis of AP2/ERF genes during fungus infection and ripening

Expression pattern of each AP2/ERF genes in *M. acuminata* was analysed using fungus infected data set of *M. acuminata*; dwarf Cavendish, genome AAA. Fungal pathogen, *Fusarium oxysporum* f. sp. cubense tropical race4 (FocTR4), infected illumina data sets were used to identify expression level of genes between banana cv ‘Brazilian’ (susceptible wild-type) and cv ‘Nongke No1’ (resistant mutant) at different time points (0h, 48h, 96h) after infection. Reads from Brazilian (susceptible) and Nongke No.1 (resistance) transcriptome data were mapped on banana genome using the Tophat software with default parameters separately and cufflinks was used for assembling the data^56^. HTseq was used to calculate number of reads mapped to a particular gene and RPKM value of mapped reads on AP2/ERF genes was calculated^57^. MeV software was used to construct the heat maps of AP2/ERF genes^58^. According to the expression level, hierarchical clustering was performed with gene samples which were shown as heat maps.

To identify the ripening related AP2\ERF genes, expression level of AP2\ERF genes in ethylene treated transcriptome data of *M. acuminata*; dwarf Cavendish, genome AAA, var. Robusta, Harichhal was analysed. Analysis was carried out using 454 transcriptome data of ethylene treated and untreated fruits^38^ to identify differentially expressing AP2/ERF genes. To validate expression levels, expression level of AP2/ERF genes in other publically available data sets were also analysed^36^. The expression profiles of genes were represented by heat maps which was generated using MEV software (http://www.tm4.org/mev.html)^58^.

### Promoter analysis and motif identification

Custom Perl script was used to extract the 1 kb upstream region of genes and this region considered as proximal promoter sequences. Plant care database^59^ was used for the identification of *cis*-regulatory elements in the promoter regions. Further, motifs present other than the AP2 domain in the protein sequences of *M. acuminata* and *M. balbisiana* were determined using the MEME software (http://meme.nbcr.net/meme/)^60^.

### Structural analysis of identified AP2/ERF proteins

Structures of AP2/ERF proteins were modelled on the basis of homology and fold recognition with default parameter using PHYRE2 server^44^. Models with higher identity were selected for detailed structural analysis. To check protein-DNA interaction, HEX molecular docking program was employed^61^. Shape and electrostatic correlation type with 3D FFT (Fast Fourier transform) mode was opted applying 0.6 value for dimensions of grid. Range of receptor and ligand molecule was set at 180 to perform global docking. Best energy conformations chose as output. Protein models and docking outputs were viewed in Chimera molecular visualization tool.

### Plant material, RNA isolation and expression analysis

Dessert (*M. acuminata)* and cooking (*M. paradisiaca)* varieties were used for gene expression analysis. *M. paradisiaca* is the hybrid of *M. acuminata* and *M. balbisiana* belonging to AAB genome^43^. Fruits from three independent plants of each cultivar at different developmental stages were harvested to collect the samples. Banana fingers from the same whorl of the hand representing similar developmental stage were treated with 100 μL/L ethylene for 24 h at 22 8C in dark and then allowed to ripen in air as described in Lohani *et al*. (2004)^33^. Other vegetative tissues were harvested from six month old banana plants growing in the field. The sampling was repeated two times at different time intervals to cater for seasonal variation. For RNA isolation, plant materials were quickly frozen in liquid nitrogen before extraction or stored at −80 ^o^C for further use.

Total RNA were isolated from banana tissues according to previously described protocol (Asif *et al*. 2006)^62^. Each RNA sample was treated with DNase I Digest kit (Sigma-Aldrich, USA) to eliminate DNA contamination. The integrity and size distribution of total RNA was analysed by agarose gel electrophoresis. A NanoQuant (Infinite® 200 PRO NanoQuant, Austria) was used for RNA quantification. DNA-free RNA (5 μg) was used for synthesis of first strand of cDNA by using Revert Aid First Strand cDNA synthesis Kit (Fermentas, USA) as per manufacturer’s recommendations. The quantitative real-time PCR expression was carried out with an ABI 7700 Sequence Detector (Applied Biosystems, USA). The transcripts were quantified by SYBR Green chemistry. The amount of cDNA was normalized by using amplification of housekeeping banana *actin* as an internal control. The data from real-time PCR amplification was estimated in terms of comparative fold expression following 2^−∆∆ct^ method. Expression analysis was carried out using three biological replicates. The list of different primers used in the study is given in Supplementary Table S3. Each reaction was performed in 20 μl (total volume) and consisted of 1X SYBR Green Master mix (Applied Biosystems, USA), 5 pmol of each primer, 1 μl cDNA template and sterile H_2_O. The steps performed during real-time PCR experiment were as follows: step (1) 50 ^o^C, 2 min; step (2) 95 ^o^C, 10 min; step (3) (95 ^o^C, 0.15 min; 60 ^o^C, 1 min) × 40 cycles.

## Additional Information

**How to cite this article**: Lakhwani, D. *et al.* Genome-wide analysis of the AP2/ERF family in *Musa* species reveals divergence and neofunctionalisation during evolution. *Sci. Rep.* **6**, 18878; doi: 10.1038/srep18878 (2016).

## Supplementary Material

Supplementary Information

## Figures and Tables

**Table 1:** Summary of AP2/ERF gene family in different plant species.

Family	Subfamily	Group	*B. rapa*	*Arabidopsis*	*O. sativa*	*P. trichocarpa*	*C. sativus*	*P. mume*	*M. acuminata*	*M. balbisiana*
AP2			30	18	29	26	20	20	46	49
ERF										
	DREB	I	15	10	9	5	5	5	14	18
		II	29	15	15	20	10	4	22	27
		III	39	23	26	35	20	20	35	44
		IV	22	9	6	6	7	6	10	10
	ERF	V	11	5	8	10	15	10	12	12
		VI	13	8	6	11	8	5	16	20
		VII	16	5	15	6	3	3	18	19
		VIII	27	15	13	17	11	10	35	39
		IX	23	17	18	42	16	18	22	25
		X	9	8	13	9	8	7	12	21
		VI-L	6	4	3	4		2	4	8
		Xb-L	9	3		4		0	0	
RAV			14	6	5	6	4	5	16	22
Soloist			1	1	1	1	4	1	3	4
**Total**			**281**	**147**	**174**	**202**	**131**	**116**	**265**	**318**

**Table 2:** Summary of newly emerged AP2/ERF genes in *Musa* A and B genomes.

*ERF Family*	*Musa accuminata*	*Musa balbisiana*	Total	New
	Total	New		
I	14	—	18	4
II	22	—	27	5
III	35	—	44	6
IV	10	—	10	—
V	12	1	12	1
VI	16	—	20	8
VII	18	1	19	2
VIII	35	1	39	4
IX	22	—	25	2
X	12	—	21	2
VI-L	4	—	8	—
RAV	16	—	22	5
AP2	46	—	49	2
Soloist	3	—	4	—
Total	265		318	
