# Supplementary material for: Genome-wide analysis of the AP2/ERF family in Musa species reveals divergence and neofunctionalisation during evolution
Source: Sci Rep. 2016 Jan 6;6:18878. doi: 10.1038/srep18878 (PMC4702079; doi:10.1038/srep18878)
Supplement: Supplementary Information [file srep18878-s1.pdf]

Supplementary information

Genome-wide analysis of the AP2/ERF family in *Musa* species reveals divergence and neofunctionalisation during evolution

Deepika Lakhwani[1,2], Ashutosh Pandey[1,$], Yogeshwar Vikram Dhar[1], Sumit Kumar Bag[1,2], Prabodh Kumar Trivedi[1,2,*], Mehar Hasan Asif[1,2,*]

[1]CSIR-National Botanical Research Institute, Council of Scientific and Industrial Research (CSIR-NBRI), Rana Pratap Marg, Lucknow-226001, INDIA

[2]Academy of Scientific and Innovative Research (AcSIR), Anusandhan Bhawan, 2 Rafi Marg, New Delhi-110 001, India

[$]Present address (AP): National Agri-Food Biotechnology Institute (NABI), Mohali-160071, Punjab, INDIA

***** Authors for correspondence:

MHA: meharasif@nbri.res.in

PKT: prabodht@nbri.res.in; prabodht@hotmail.com

Running title: AP2/ERF gene family in *Musa* species

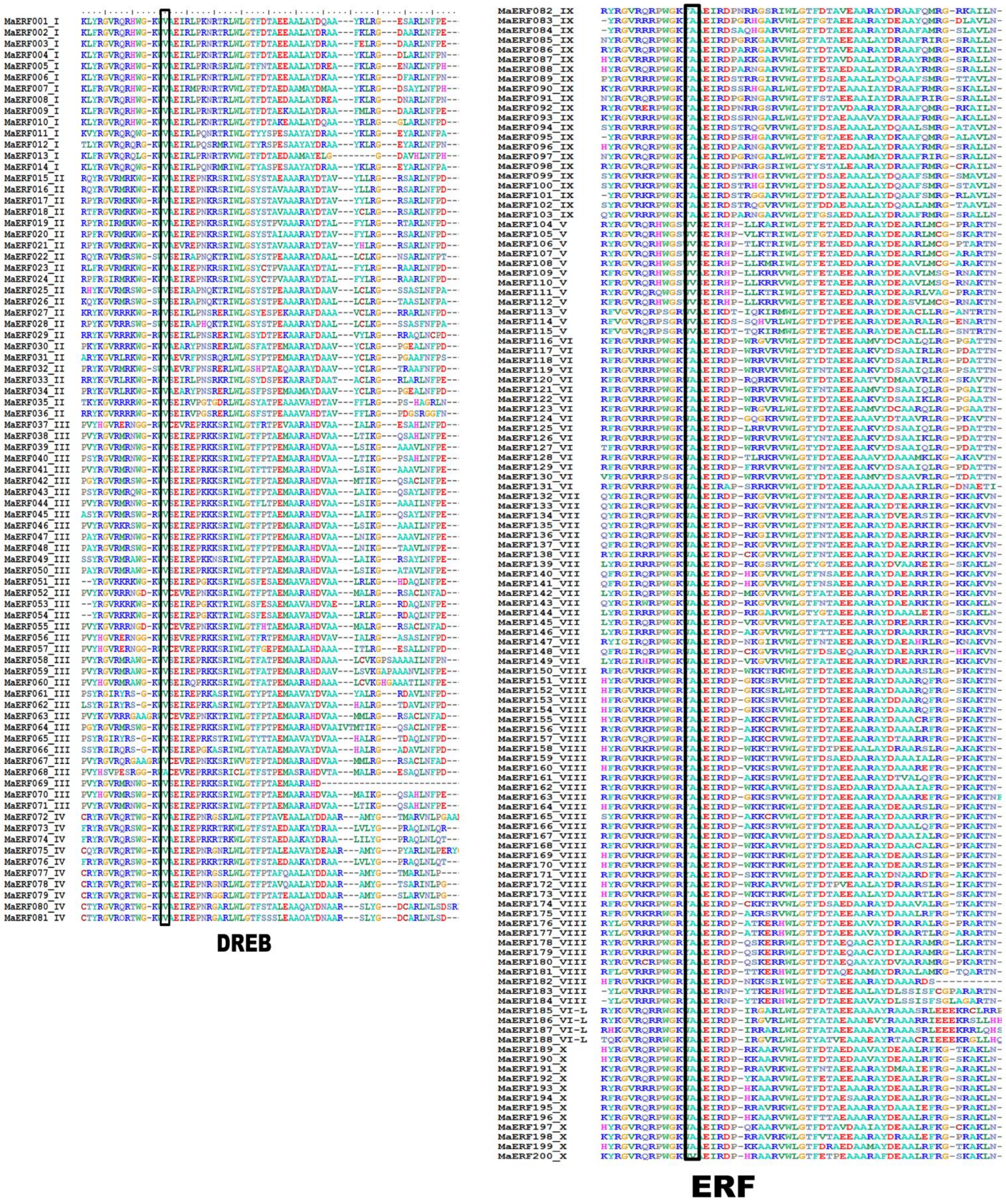

Figure S1. Alignment of amino acid sequences of AP2/ERF proteins of *M. acuminata*. Alignment of different groups of proteins show conserved amino acids at 14th and 19th position of DREB and ERF subfamily. White letters with black background indicate amino acids conserved pattern in ERF proteins. Red blocks show the amino acid conservation at 14th and 19th position in both groups.

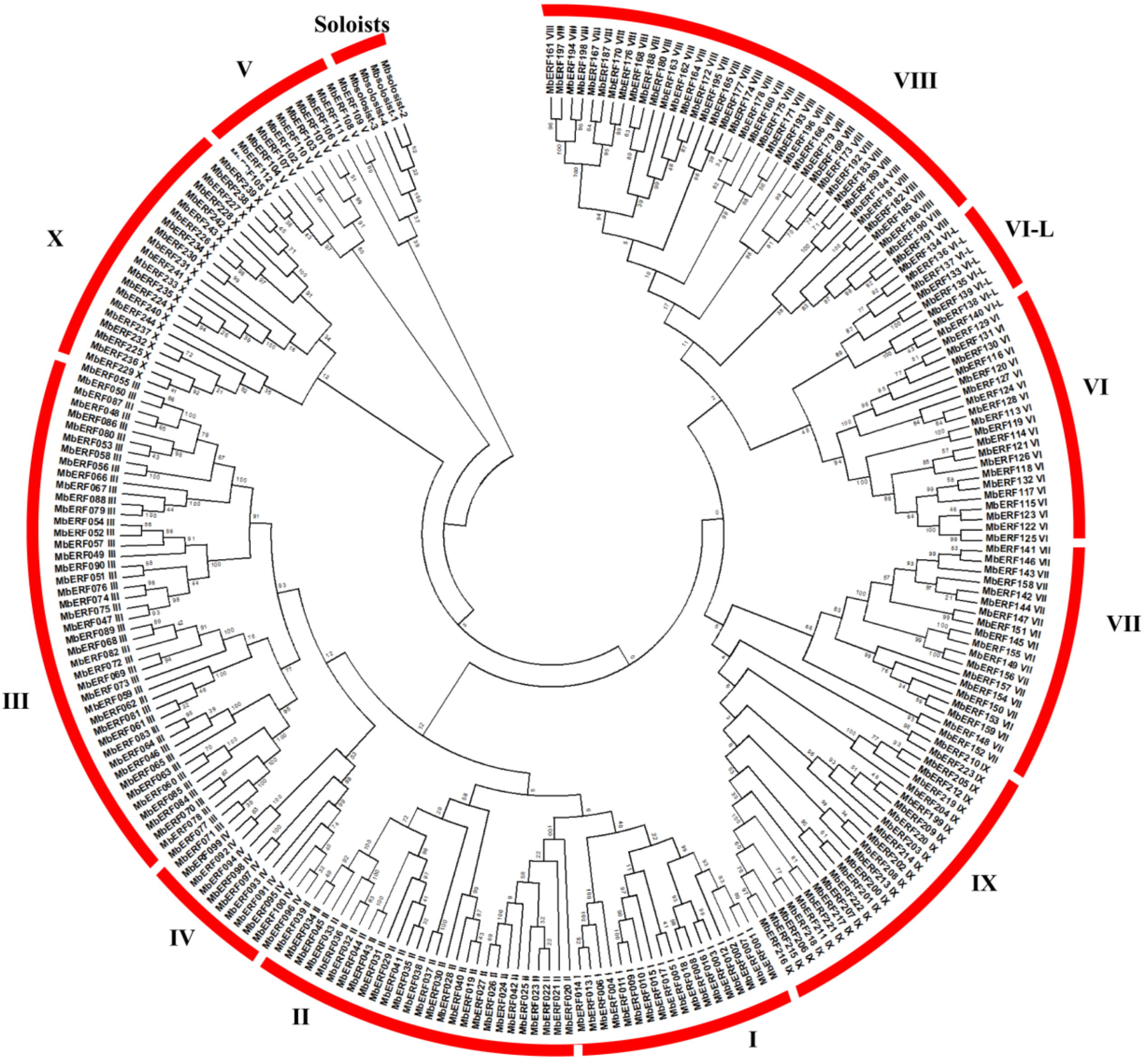

Figure S2. Phylogenetic tree of AP2/ERF proteins of *M. balbisiana*. AP2/ERF genes classified into different groups and showed with broken lines.

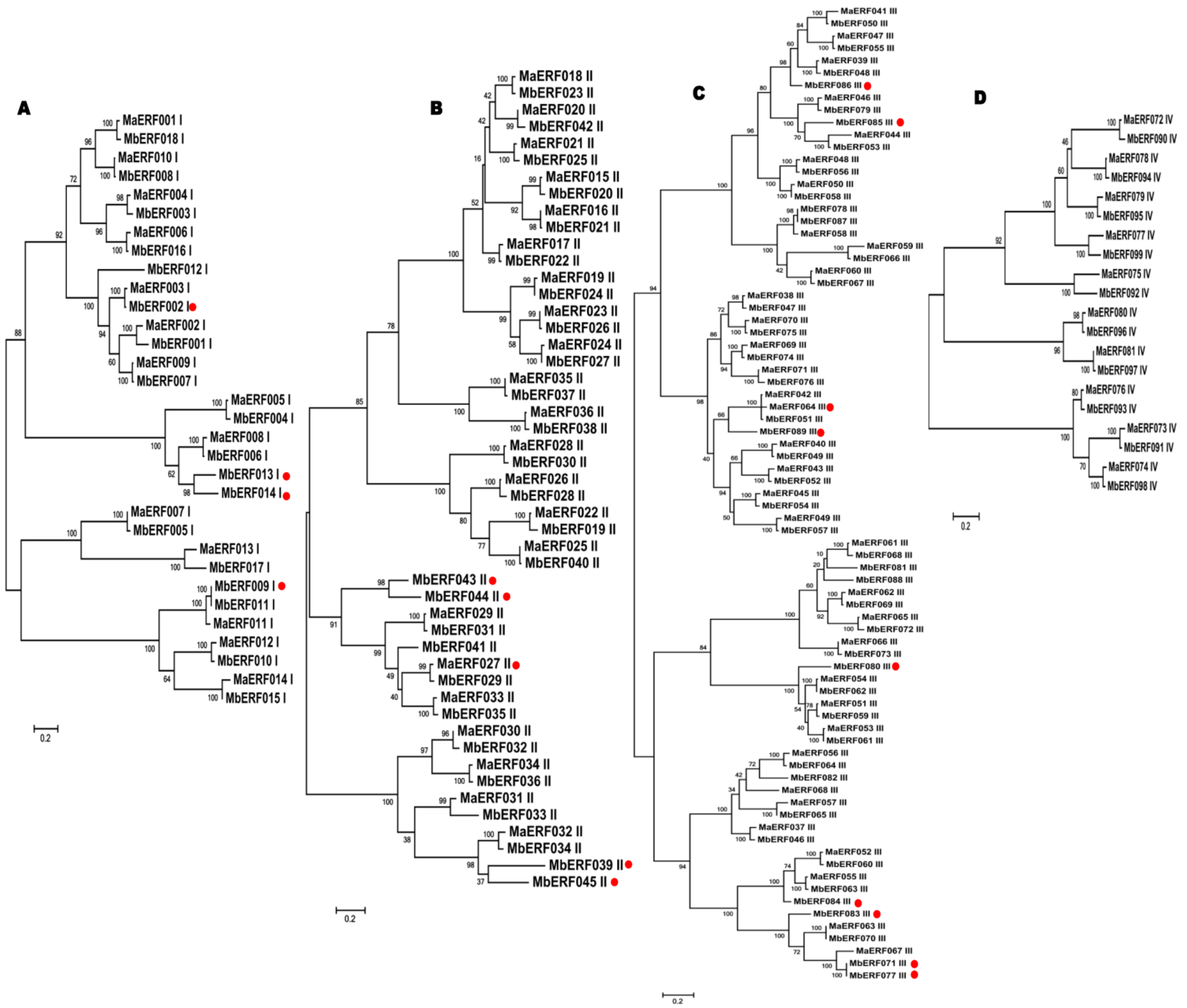

Figure S3. Phylogenetic tree of DREB subfamily proteins of *M.acuminata* and *M.balbisiana*. Proteins of each group in DREB class are align with each other separately and construct tree using 500 bootstrap value. Each Tree belongs to different groups.

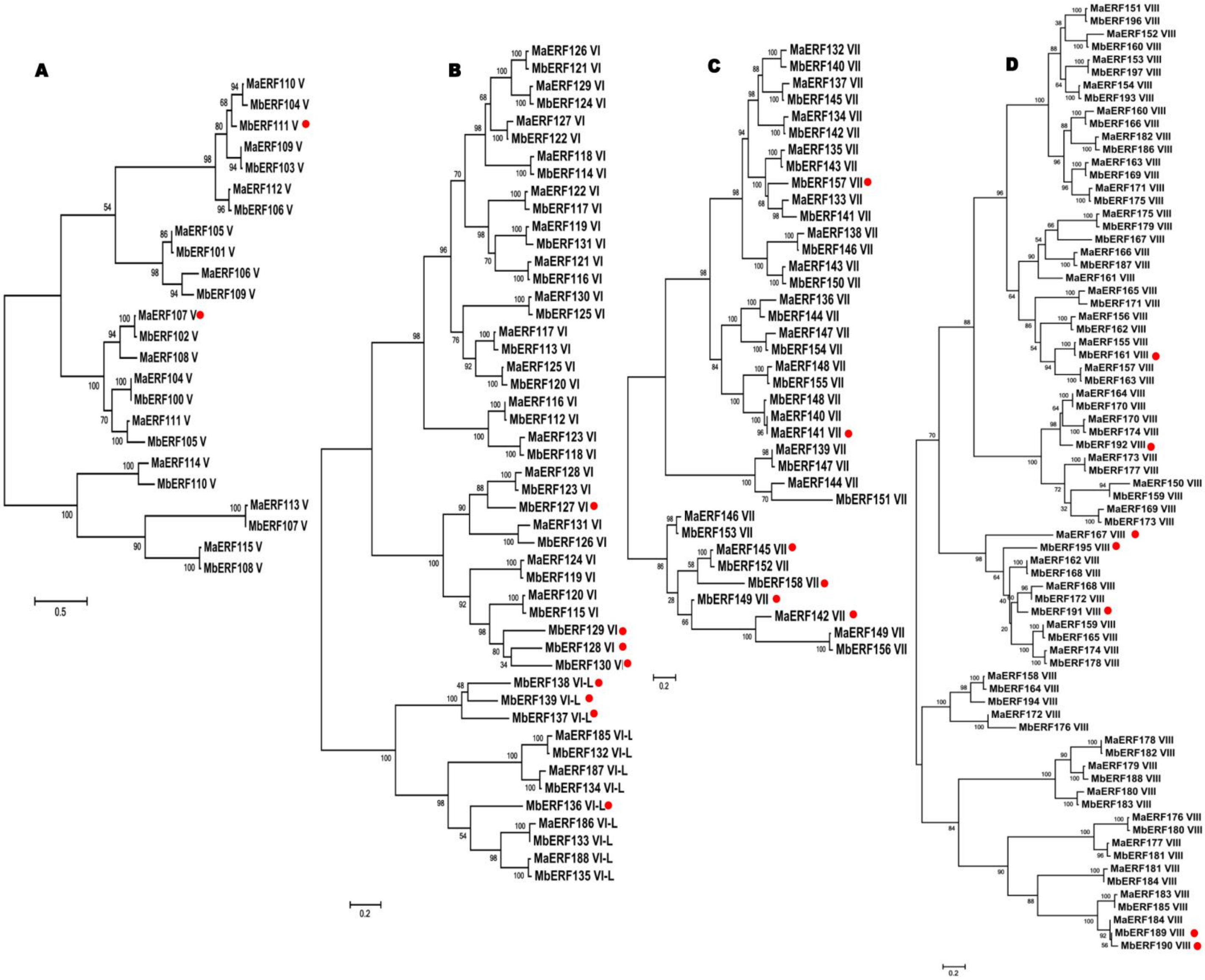

Figure S4. Phylogenetic analysis of ERF subfamily proteins of *M.acuminata* and *M.balbisiana*. ERF proteins of group V to VIII in ERF subfamily are aligned with each other separately and construct tree using 500 bootstrap value. Each Tree belongs to different groups.

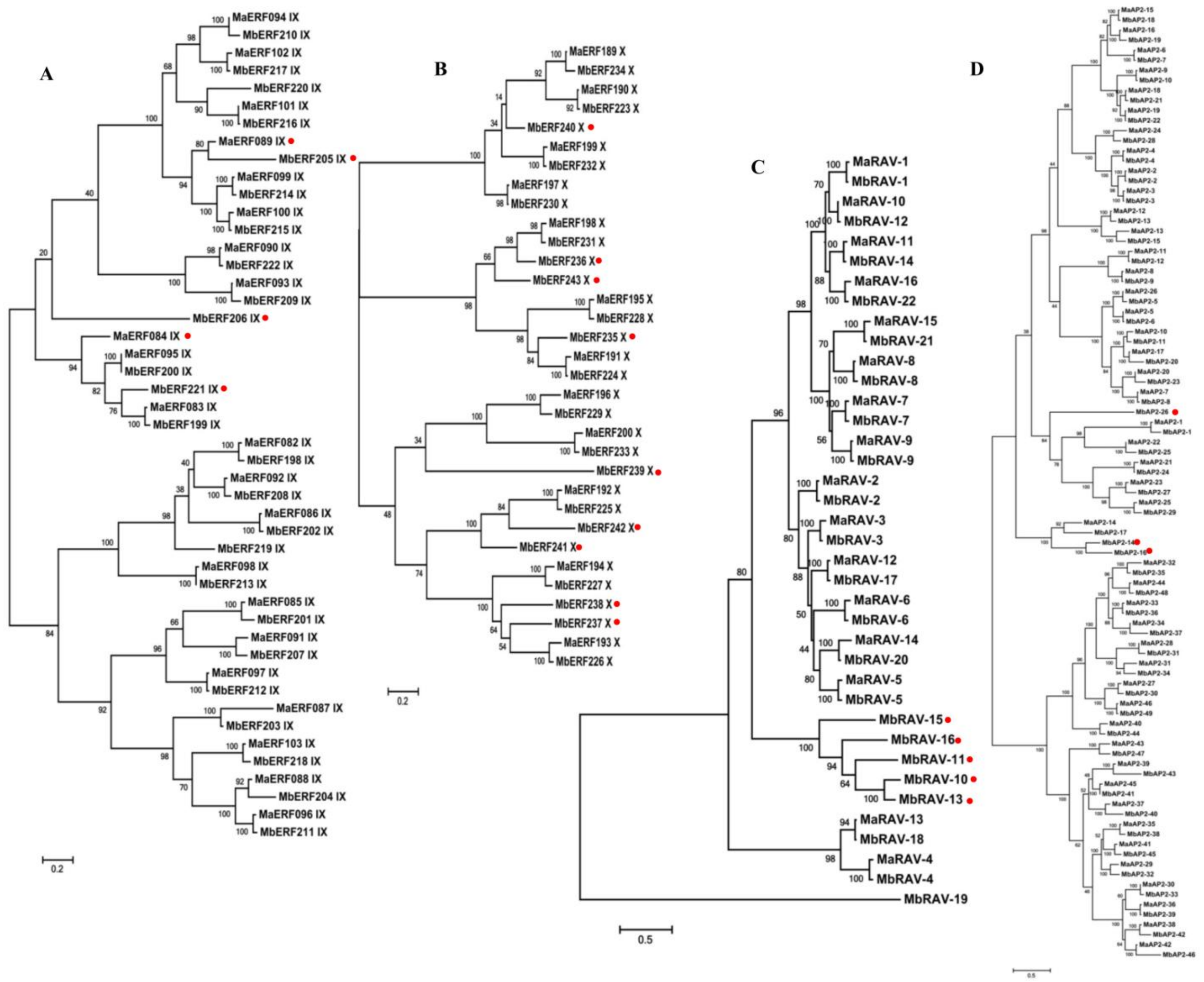

Figure S5. Phylogenetic tree of proteins of ERF subfamily, RAV and AP2 family. Each tree shows the evolutionary relationship of genes within family.

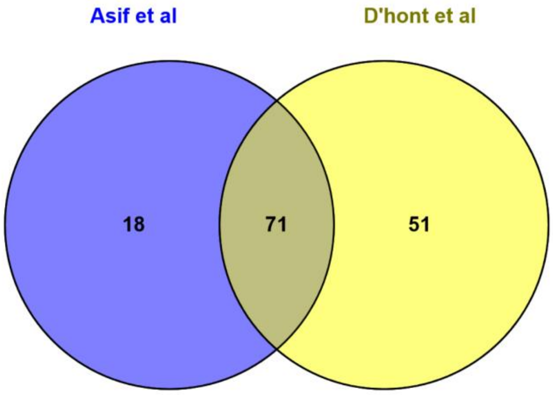

Figure S6. Venn diagram of differentially expressing genes obtained from RNA-seq data.

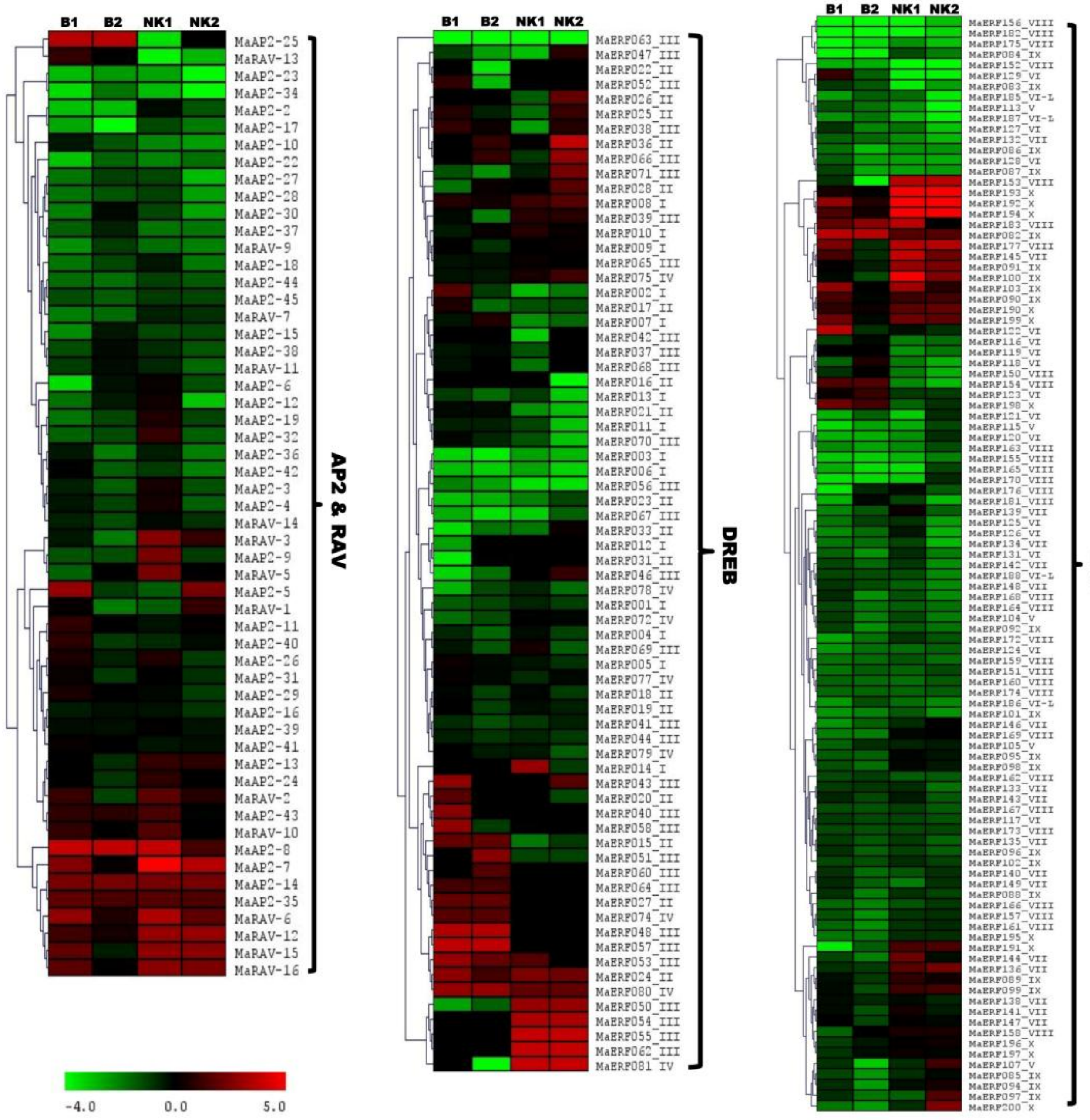

Figure S7. Heat-map showing the expression pattern of AP2/ERF genes in response to fungus in the root of banana. Fold change value used to show the differential expression profile of ERF genes in the root of resistant (BK) and susceptible (NK) varieties after infection of fungus at two time points 48h and 90h.

Table S1. Conserved motifs present in ERFs in *Musa acuminata*.

Groups name	Motifs	
**Group I**	WDE, KYPS	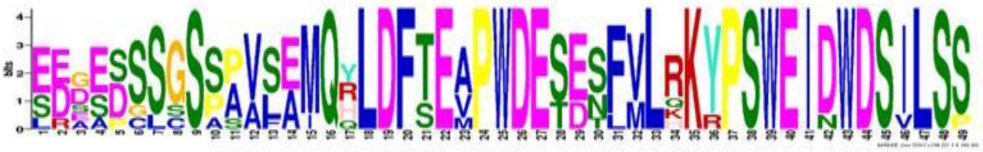
	LNFP	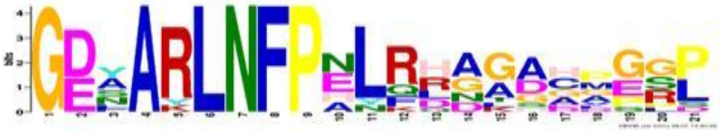
**Group II**	EAR motif	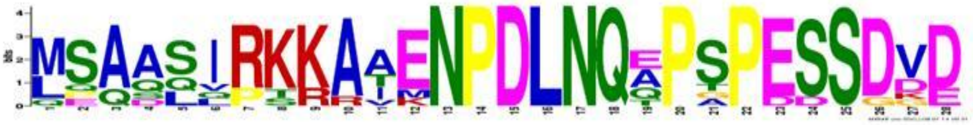
	LNFP	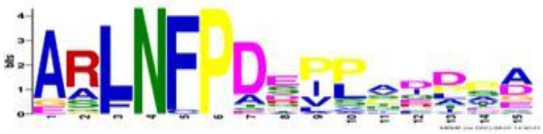
	LWSF	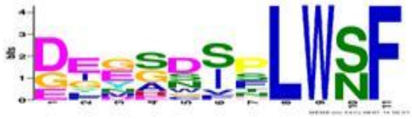
**Group III**	LPR[P/A]	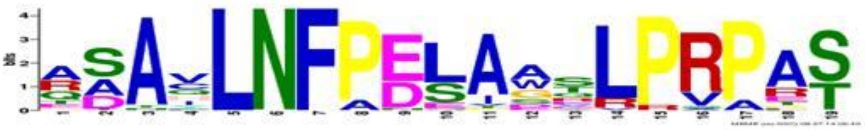
	D[I/V]QAA	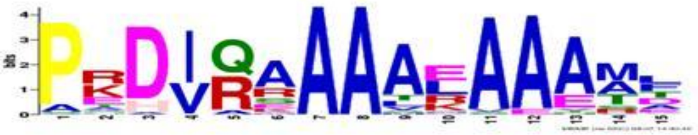
		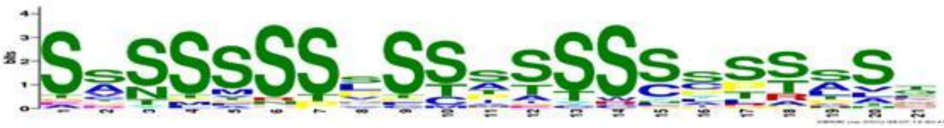
**Group IV**	LPRPS	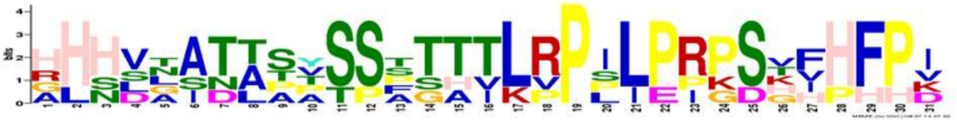
**Group V**		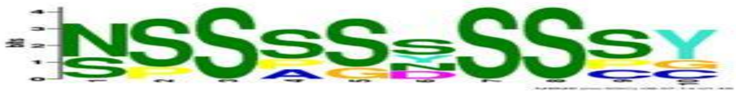
	TNF	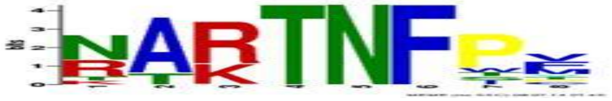

**Group VI**	SP[T/V]SVL	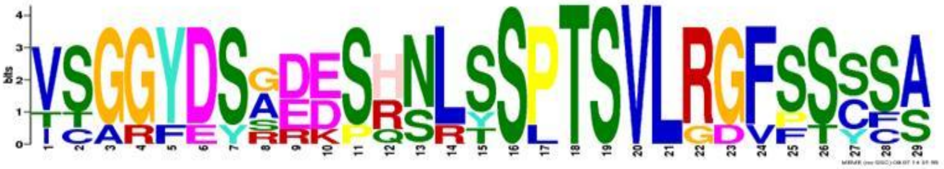
	DLFP	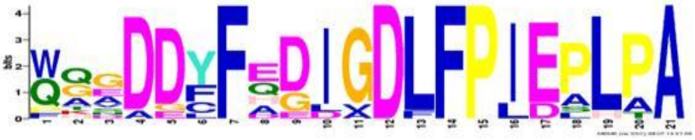
	TNF	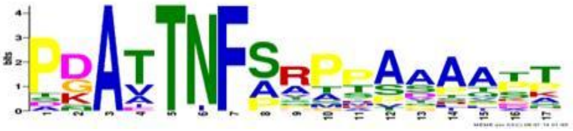
**Group VII**	MCGGAI[I/L]	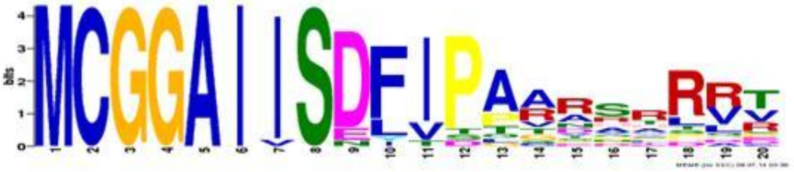
	DFEADF	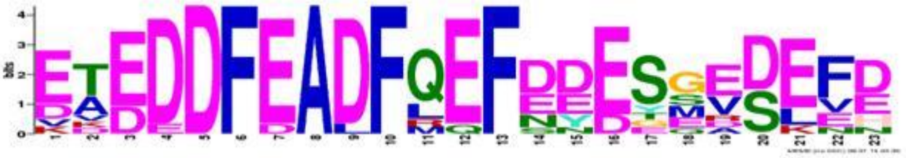
	NFP	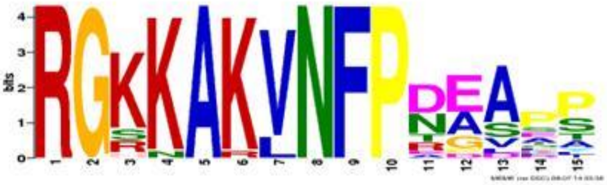
	LWS	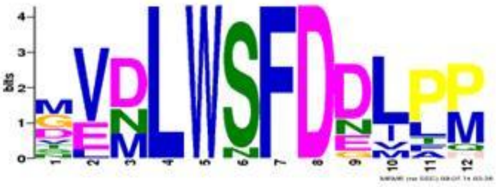
	SD[E/Q/R]GSNSF	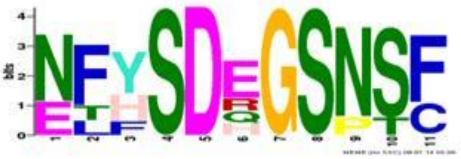
	LWP	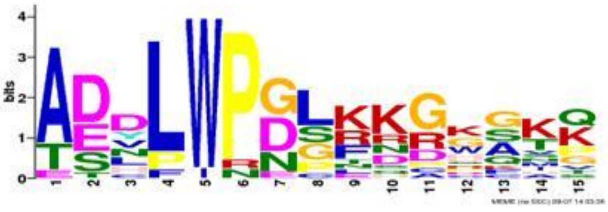

**Group VIII**	TNF	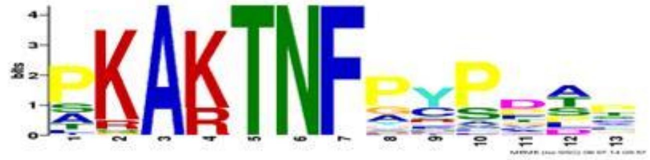
	EAR motif	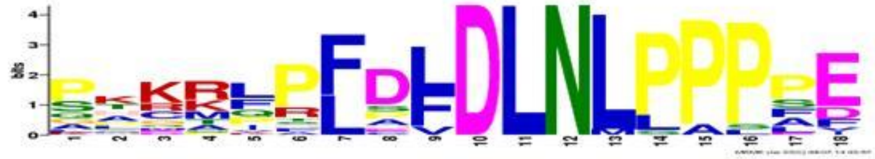
		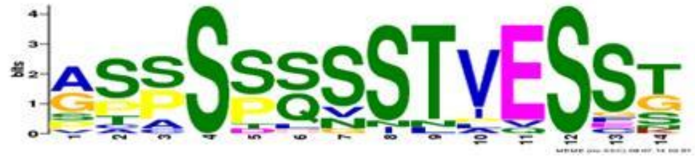
**Group IX**	LNFP	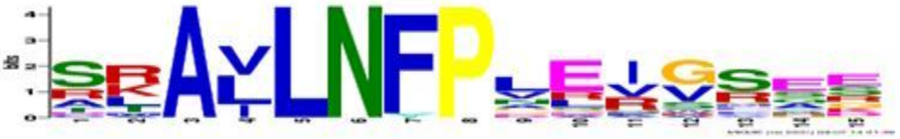
**Group X**		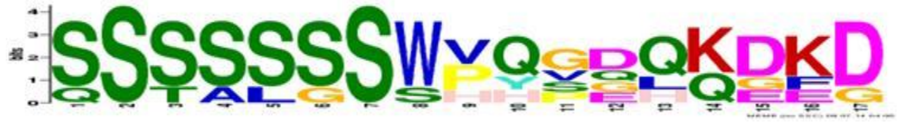

Table S2. Conserved motifs present in ERFs in *Musa balbisiana*.

Groups name	Motifs	
Group I	LNFP	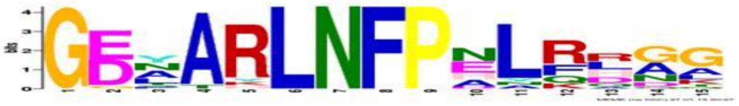
	WEID, KYPS	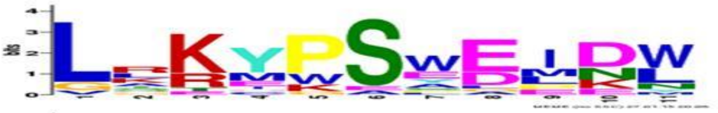
		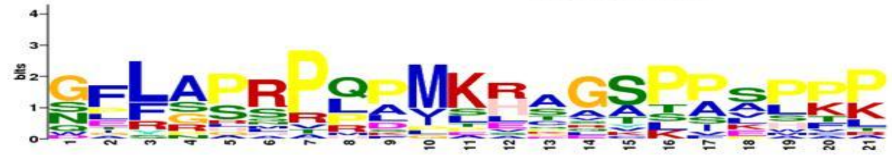
		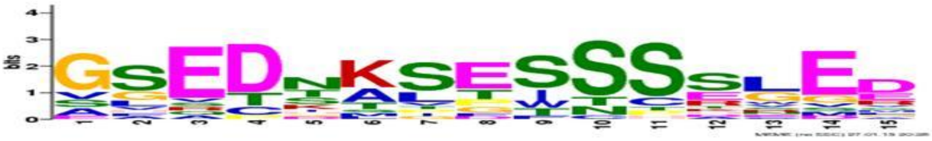
Group II	EAR motif	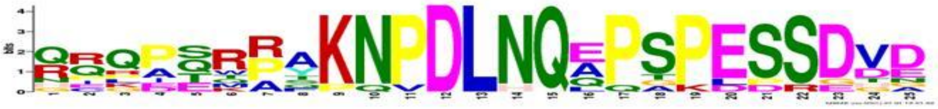
	LWSF	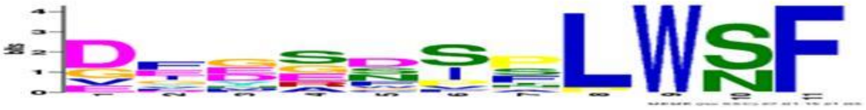
		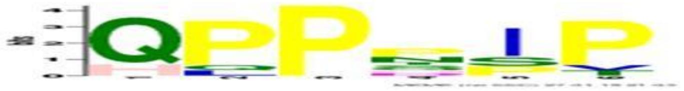
		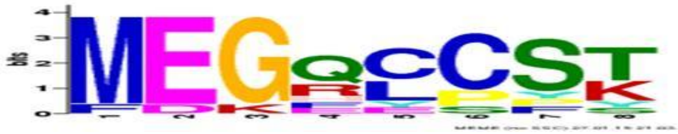
Group III	LPR[P/A]	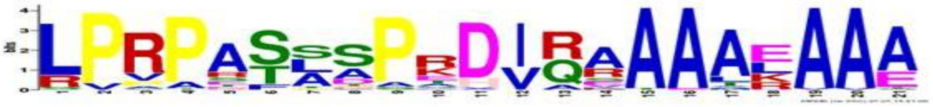
		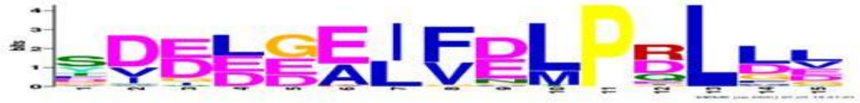
		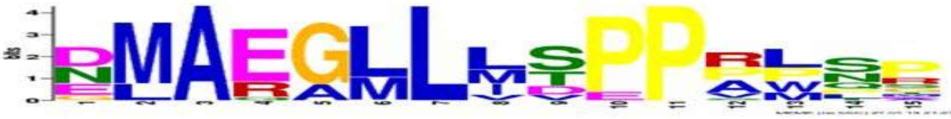
	LWSY	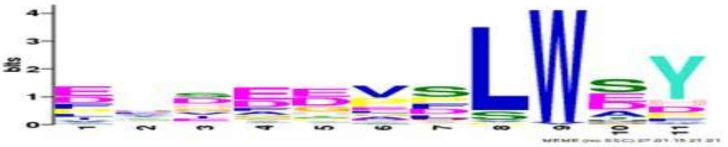
		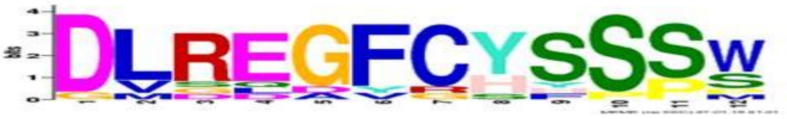

Group IV		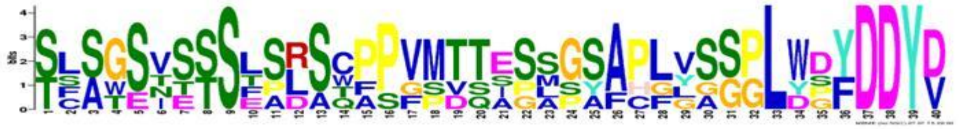
		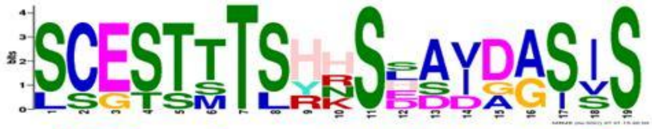
		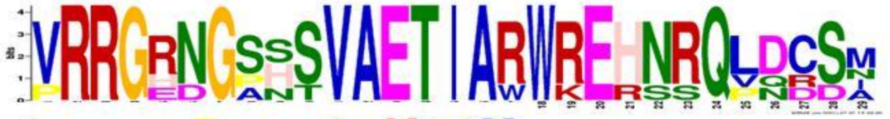
		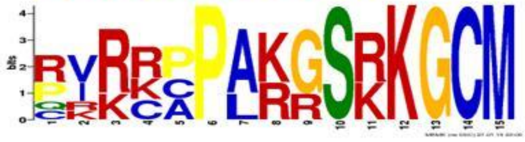
Group V		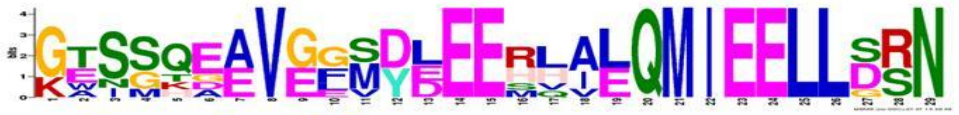
		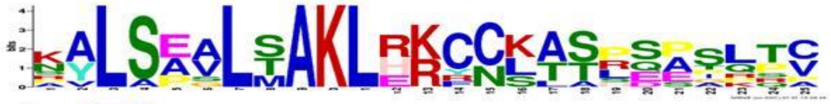
		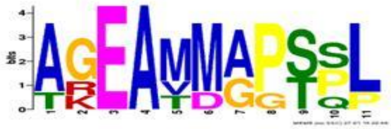
		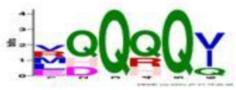
Group VI		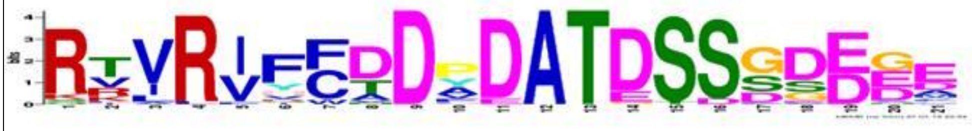
		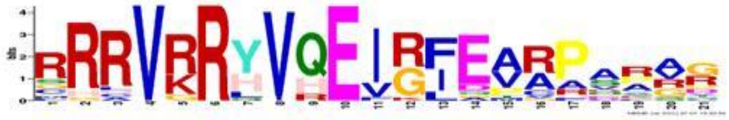
	SP[T/V]SVL	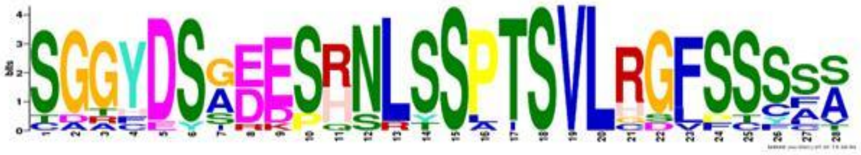
		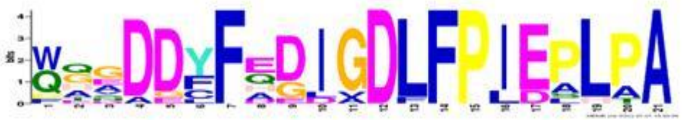

**Group VII**	MCGGAI[I/L]	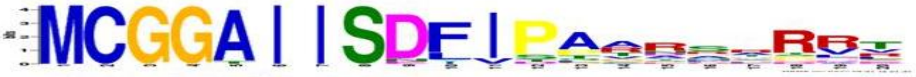
	NFP	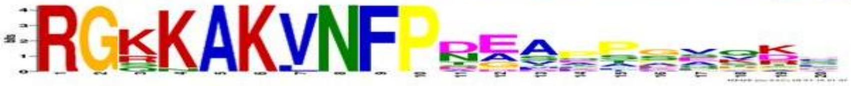
	DFEAD	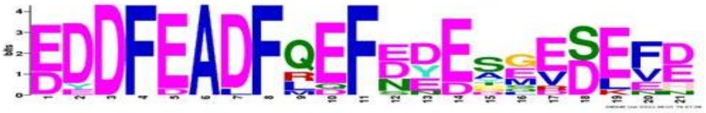
	LWS	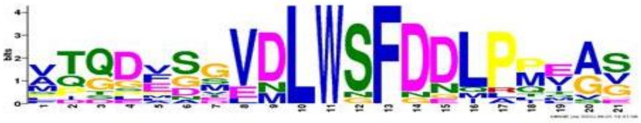
	SD[E/Q/R]GSN SF	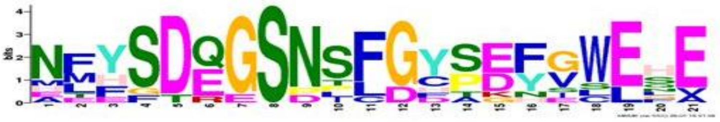
		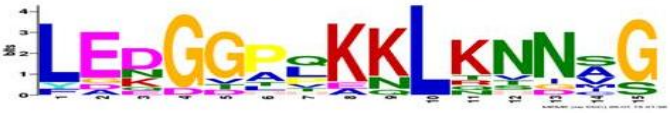
		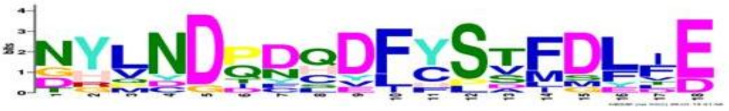
**Group VIII**	EAR motif	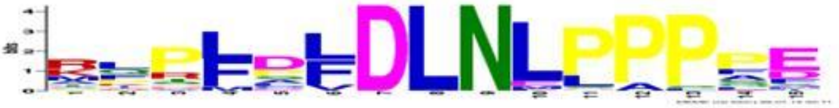
	TNF	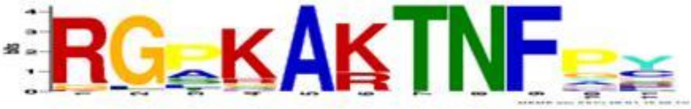
		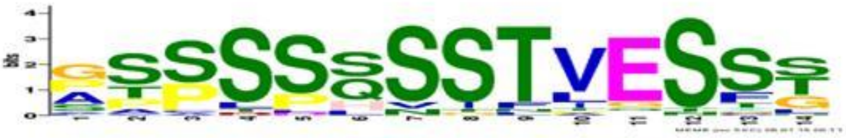
		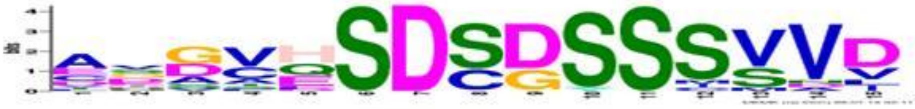
		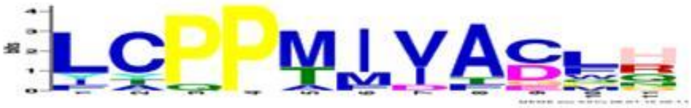
		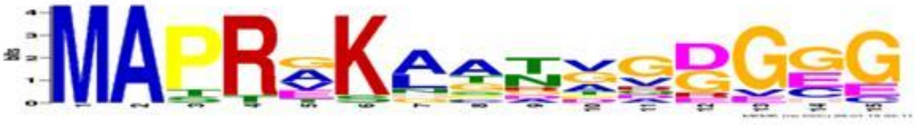
	DLNL	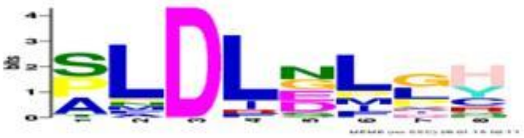

Table S3. List of Primers used in qRT PCR.

S.no	Gene Name	Forward Sequence	Reverse Sequence
1	MaERF182	CCTCGCCCGATATCAACCTC	GCGATCTACGACGGAGGAAG
2	MaERF025	AGGCACTACAAGGGTGTGAGGATG	TGCCTTTCAAGCAGAGCACCGCTGC
3	MaERF022	TCTCAGAGATCAGAGCACCGAACCAG	GGACATGGTGGTGTCCGGAAGCTGAAT
4	MaERF083	ATCAGAAGGAGGGCGGCGAGACCAAG	GGCGGTGCCGAAGGTGCCGAGCCAGA
5	MaAP235	ATACTCGAAGGTGCCGGTGGTGG	CATGGCGGGTGCACCTACCTGCCGT
6	MaERF173	TGTGAGGAAGCGGCCGTGGGGAAGG	CTTGGCCTTGGACCCACGGAGCGT
7	MaERF161	ACACCGTCGAGTCCTCCACCCCTTC	ACACCGTCGAGTCCTCCACCCCTTC
8	MaERF187	AGTAGTGGGAAGTTGGAGAGGTCG	GGTCCACGTGGATCTCCCGGACC
9	MaAP2-44	AGGAGGACAGCTGCTGCCTAATGG	GCCGGAGTCGTCGTTCTCCACCTCT
10	MaAP2-23	ATGGCGGCGACAACGGGAAGCGGAG	AGCCTCCTCGGCATCGTAAGCAC
11	MaERF026	GTACAAGGGTGTGAGGATGAGGAGC	GCAGAGCAAGGCCGCATCGTAGGCT
12	MaAP2-13	ATCCAGGATCTCCGCGCGGTCGACG	CTTCCGGTCCACCTGTGCCTGGT
13	MaRAV6	TGTCGTCGTCTCAGTACAAAGGCG	GGCTCGCTTGTCGGAGAGAGGCT
14	MaAP2-46	ACGTGGACGGGATGGCGCCGGAGCTG	ACGCCCTTGCTGCTGCATGCGCAGT
15	GSMUA_Achr2T16160_001 (ACT101)	ATGACATGGAAAAGATCTGGCA	CCTGAATGGCAACATACATAGC
